# Ploidy effect and genetic architecture exploration of stalk traits using DH and its corresponding haploid populations in maize

**DOI:** 10.1186/s12870-016-0742-3

**Published:** 2016-02-25

**Authors:** Yujie Meng, Junhui Li, Jianju Liu, Haixiao Hu, Wei Li, Wenxin Liu, Shaojiang Chen

**Affiliations:** National Maize Improvement Center of China, China Agricultural University (West Campus), 2# Yuanmingyuan West Road, Beijing, 100193 China; Beijing Key Laboratory of Crop Genetic Improvement, College of Agronomy, China Agricultural University (West Campus), 2# Yuanmingyuan West Road, Beijing, 100193 China; Institute of Plant Breeding, Seed Science, and Population Genetics, University of Hohenheim, 70599 Stuttgart, Germany

**Keywords:** Maize, Ploidy effect, Rind penetrometer resistance, In vitro dry matter digestion, DH, Haploid population

## Abstract

**Background:**

Doubled haploid (DH) lines produced via in vivo haploid induction have become indispensable in maize research and practical breeding, so it is important to understand traits characteristics in DH and its corresponding haploids which derived from each DH lines. In this study, a DH population derived from Zheng58 × Chang7-2 and a haploid population, were developed, genotyped and evaluated to investigate genetic architecture of eight stalk traits, especially rind penetrometer resistance (RPR) and in vitro dry matter digestion (IVDMD), which affecting maize stalk lodging-resistance and feeding values, respectively.

**Results:**

Phenotypic correlation coefficients ranged from 0.38 to 0.69 between the two populations for eight stalk traits. Heritability values of all stalk traits ranged from 0.49 to 0.81 in the DH population, and 0.58 to 0.89 in the haploid population. Quantitative trait loci (QTL) mapping study showed that a total of 47 QTL for all traits accounting for genetic variations ranging from 1.6 to 36.5 % were detected in two populations. One or more QTL sharing common region for each trait were detected between two different ploidy populations. Potential candidate genes predicated from the four QTL support intervals for RPR and IVDMD were indirectly or directly involved with cellulose and lignin biosynthesis, which participated in cell wall formation. The increased expression levels of lignin and cellulose synthesis key genes in the haploid situation illustrated that dosage compensation may account for genome dosage effect in our study.

**Conclusions:**

The current investigation extended understanding about the genetic basis of stalk traits and correlations between DH and its haploid populations, which showed consistence and difference between them in phenotype, QTL characters, and gene expression. The higher heritabilities and partly higher QTL detection power were presented in haploid population than in DH population. All of which described above could lay a preliminary foundation for genetic architecture study with haploid population and may benefit selection in haploid-stage to reduce cost in DH breeding.

**Electronic supplementary material:**

The online version of this article (doi:10.1186/s12870-016-0742-3) contains supplementary material, which is available to authorized users.

## Background

Maize (Zea mays L.) is one of the important grain and feed crops in which the stalk, as one indispensable part of plant morphology, serves as the conductor of transporting water and nutrients. Stalk lodging lead to yield losses estimated to range from 5 to 20 % annually worldwide [1]. Rind penetrometer resistance (RPR), which is one of the reliable indicators of stalk strength, has been widely used to measure stalk strength and improve stalk lodging resistance [2, 3]. Maize is also one of the most important annual forage crops. In vitro dry matter digestion (IVDMD) has been the most useful evaluating indicator for maize forage variety examination in many European countries [4]. Therefore, a further and better understanding of the molecular basis for RPR and IVDMD is crucial for breeding lodging-resistant and highly digestible maize [5].

The genetic analysis of quantitative traits is difficult and complex in maize, and quantitative traits are affected by key genes and interacting networks of small-effect genes. Therefore, different studies have provided different results including quantitative trait loci (QTL) number, distribution, and genetic effects for one trait [6, 7]. This lack of conformity may also be explained by the many differences in parental materials, segregation-population types, ecological conditions, genetic maps, analytical methods and phenotype evaluation [8, 9]. Moreover, high genome dosage levels have effect on genetic analysis [10, 11].

Due to the advantages of time-saving and high genetic variance, doubled haploid (DH) technology is routinely used in modern maize breeding for production of homozygous parental lines for maize hybrid breeding and constructing DH populations for genetic research [12–15]. Although haploid populations possess the characteristics of genetic homozygosity and have one genome dosage, moderate to strong correlations have been identified between small size DH populations and their haploid version populations for some agronomic traits [16]. Moreover, haploid lines could react more sensitively to biotic and abiotic stresses and, therefore, they would effectively uncover susceptibility to diseases and environmental constraints. In *A. thaliana*, the utility and power of haploid genetics had been reported. Haploids can provide genetic analysis advantages that are not available in diploids, such as specifically pyramiding multiple mutant combinations, forward mutagenesis screens and swapping of nuclear and cytoplasmic genomes [17]. In yeast, haploid screens represent an ideal platform for negative selection since a certain genetic lesion set by mutagenesis will exert equal effects in all cells [18]. In this regard, the haploid lines may also be interesting in the genetic architecture exploration of maize quantitative traits.

Different segregating populations have been used in linkage analysis or genome-wide association study of RPR, and the genome set number of all these populations was two. The results suggested the genetic complexity of RPR. Flint Garcia et al. [19] first detected 35 RPR QTL in four F_2:3_ populations, which accounted for more than 33 % of the total phenotype variation. Hu et al. [20] detected 9 QTL in a RIL population developed from the cross of B73 × Ce3005, which could explain 1.15–12.43 % of the phenotypic variation. Li et al. [21] narrowed the QTL interval which had the largest effect among the 7 QTL of RPR detected in two RIL populations by the method of haplotype analysis. Peiffer et al. [22] reported that 18 family-nested QTL and 141 significant GWAS associations were identified for RPR across NAM (nested association mapping) and IBM (intermated B73 × Mo17) families, while numerous weak associations were found in the NCRPIS (North Central Regional Plant Introduction Station) diversity panel for RPR. Mutations, *brittle stalk (BK)* genes exhibiting a lower proportion of cellulose, had dramatically weakened tissue mechanical strength than that of wild type stalks [23].

Moreover, whole plant digestibility, which can reflect the feeding value, has been extensively studied in forage maize, and several reports of QTL analyses with low-density markers for stalk digestibility in forage maize were published [24, 25]. Maize mutants and/or genetically engineered plants have highlighted a few genes affecting maize cell wall degradability [26, 27]. Reports have emerged on nucleotide diversity and the extent of linkage disequlibrium (LD) at the gene locus of lignin and cellulose synthesis [28–30].

It was well known that plant breeders are desired to choose lines based on minimizing negative effects of genotype agronomic value, so it was crucial to perform research on the genetic architecture of stalk traits, especially for RPR and IVDMD. In this study, we first used a DH population combined with the corresponding haploid population to identify QTL and observe candidate gene expression about stalk traits. Our objectives were to: (1) explore the genetic architecture of stalk traits; (2) evaluate consistence and difference in phenotype, QTL characters, and gene expression between two different ploidy populations in stalk traits; and (3) preliminary propose and illustrate a ploidy effect mechanism for RPR and IVDMD under one genome dosage situation with the QTL mapping method.

## Results

### Performance of parental lines, F1 generation and DH and haploid populations derived from each DH line

**Fig. 1:**
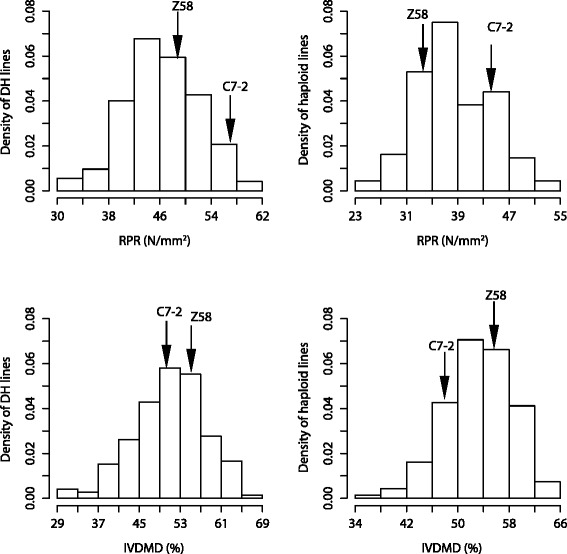
Frequency distribution of RPR and IVDMD for lines in two different ploidy populations. Parental strain values were indicated with *arrows*

Performance of parents and derived DH and haploid populations across five environments was presented in Table 1. RPR, water content (WC), acid detergent fiber (ADF), neutral detergent fiber (NDF), and cellulose(Cel) of the male parent Chang7-2 (C7-2) showed significantly higher values than those of the female parent Zheng58 (Z58) in both DH and haploid populations. In contrast, for IVDMD and WSC (water soluble carbohydrate), Z58 had a higher value than the male parent C7-2 in both populations. There was no significant difference in lignin (Lig) content between two parents in the DH and haploid populations. RPR and IVDMD showed a normal distribution in both two ploidy populations (Fig. 1). For all traits investigated in this study, coefficients of variation (CV) in the DH and haploid population ranged from 7.56 to 49.48 % and from 8.28 to 35.28 %, respectively. The genotypic variance (*σ*_*G*_^2^) was significant at *P* < 0.01 in both the DH and haploid populations (Table 2). The broad-sense heritability (*h*_*B*_^2^) of all traits in the DH population were intermediate to high (0.49<*h*_*B*_^2^<0.81) as well as in the haploid population (0.58<*h*_*B*_^2^<0.89). Notably, *h*_*B*_^2^for all traits were higher in the haploid population than in the DH population except for WC, of which *h*_*B*_^2^ was slightly lower in haploid population (0.58) than in DH population (0.60).

**Table 1:** Phenotypic performance of all stalk traits in DH and haploid populations

Trait	Unit	Population Ploidy	Z58 (mean ± SD^a^)	C7-2 (mean ± SD)	t test^b^	PM^c^	F_1_ plants/8	PA^d^	CV (%)^e^
RPR	N/mm^2^	DH	49.45 ± 4.09	56.68 ± 4.23	*	53.07	46.32 ± 4.37	46.32 ± 5.76	24.17
		Haploid	32.67 ± 3.71	43.34 ± 3.62	**	38.01		38.60 ± 5.79	26.38
IVDMD	%	DH	55.02 ± 3.97	49.92 ± 4.04	NS	52.47	56.42 ± 4.55	50.83 ± 6.83	21.66
		Haploid	56.07 ± 2.40	46.07 ± 2.33	**	51.07		53.19 ± 5.29	13.50
WC	%	DH	73.12 ± 2.50	78.73 ± 2.58	**	75.93	75.54 ± 2.70	75.36 ± 2.51	7.56
		Haploid	69.05 ± 2.58	78.25 ± 2.50	**	73.65		70.59 ± 2.61	8.28
ADF	%	DH	30.23 ± 3.46	37.37 ± 3.52	**	33.8	29.35 ± 3.70	34.75 ± 5.14	14.70
		Haploid	26.70 ± 2.60	36.78 ± 2.54	**	31.74		28.15 ± 4.70	16.70
NDF	%	DH	48.86 ± 4.79	60.41 ± 4.86	**	54.64	47.04 ± 5.44	55.02 ± 7.24	13.15
		Haploid	45.09 ± 3.42	62.44 ± 3.36	**	53.76		49.70 ± 6.07	12.22
Lig	%	DH	7.83 ± 1.03	7.74 ± 1.05	NS	7.79	9.75 ± 1.20	8.64 ± 1.15	13.31
		Haploid	8.84 ± 1.00	8.59 ± 0.97	NS	8.71		9.44 ± 1.45	15.32
Cel	%	DH	24.64 ± 2.45	29.68 ± 2.49	**	27.16	22.05 ± 2.72	26.89 ± 3.46	24.36
		Haploid	22.52 ± 2.33	30.83 ± 2.28	**	26.67		24.42 ± 3.34	25.67
WSC	%	DH	25.08 ± 4.52	21.22 ± 4.60	NS	23.15	25.20 ± 4.98	23.35 ± 5.31	49.48
		Haploid	25.91 ± 3.40	16.22 ± 3.31	**	21.06		25.73 ± 5.24	35.28

^a^ Standard deviation

^b^* Significant at *P* < 0.05, ** Significant at *P* < 0.01, *NS* not significant

^c^ Means of two parental lines

^d^ Population average of traits

^e^ Coefficient of variation

**Table 2:** Variance components and broad-sense heritability (*h*_*B*_^2^) of all stalk traits in DH and haploid populations

Traits	Unit	DH population				Haploid population			
		*σ*_*G*_^2^	*σ*_*G* × *E*_^2^	*σ*_*e*_^2^	*h*_*B*_^2^	*σ*_*G*_^2^	*σ*_*G* × *E*_^2^	*σ*_*e*_^2^	*h*_*B*_^2^
RPR	N/mm^2^	23.41**	6.80**	75.80	0.72	23.88**	0.66^NS^	51.28	0.87
IVDMD	%	34.31**	15.31**	51.38	0.81	18.36**	3.83**	26.77	0.89
WC	%	3.41**	2.80**	17.62	0.60	2.37**	2.77**	17.48	0.58
ADF	%	18.82**	10.52**	33.34	0.78	14.63**	6.43**	20.22	0.85
NDF	%	37.05**	24.29**	60.38	0.77	21.90**	14.92**	32.90	0.81
Lig	%	0.56**	1.26**	3.45	0.49	0.96**	0.29^NS^	4.45	0.73
Cel	%	8.17**	3.99**	18.77	0.75	6.75**	0.77^NS^	16.07	0.85
WSC	%	16.23**	11.50**	61.00	0.66	13.31**	3.81*	46.69	0.77

* Significant at *P* < 0.05, ** Significant at *P* < 0.01, *NS* not significant

### Inter-population and intra-population phenotypic correlation

**Fig. 2:**
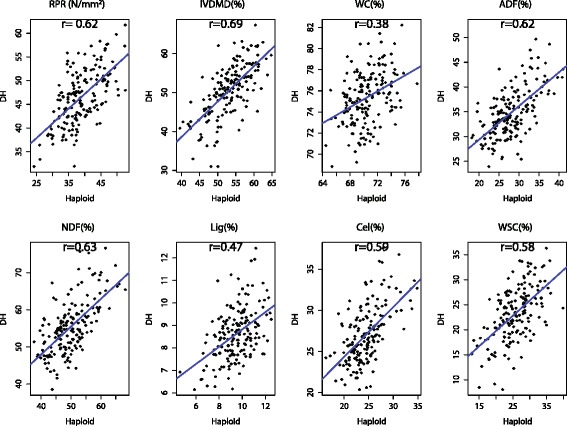
Phenotypic correlations of stalk traits between DH and haploid populations. BLUEs of the haploid population were presented in *x axis* and BLUEs of the DH population were presented in *y axis*

The phenotypic correlation coefficients of all stalk traits between the DH and haploid populations ranged from 0.38 to 0.69 (Fig. 2). Coefficients of phenotypic correlation among different traits in DH population showed similar patterns to those in haploid population. In both populations, ADF, NDF and Cel showed high positive correlation among themselves, significantly positively correlated with RPR but negatively correlated with IVDMD, Lig and WSC. RPR negatively correlated with IVDMD but with different correlation coefficients in DH and haploid populations, respectively (Table 3).

**Table 3:** Phenotypic correlations among stalk traits. Correlation coefficients among stalk traits in DH population and haploid population were shown in upper and lower triangular cells, respectively

Trait	RPR	IVDMD	WC	ADF	NDF	Lig	Cel	WSC
RPR		−0.31**	−0.25**	0.37**	0.32**	−0.07^NS^	0.35**	−0.22**
IVDMD	−0.51**		−0.39**	−0.92**	−0.93**	0.25**	−0.88**	0.87**
WC	−0.004^NS^	−0.20**		0.37**	0.44**	−0.34**	0.32**	−0.44**
ADF	0.49**	−0.83**	0.32**		0.97**	−0.37**	0.95**	−0.84**
NDF	0.41**	−0.85**	0.36**	0.93**		−0.46**	0.93**	−0.87**
Lig	0.02^NS^	0.34**	−0.45**	−0.48**	−0.59**		−0.34**	0.32**
Cel	0.44**	−0.72**	0.29**	0.84**	0.78**	−0.33**		−0.77**
WSC	−0.31**	0.78**	−0.34**	−0.78**	−0.81**	0.41**	−0.61**	

** Significant at *P* < 0.01, *NS* not significant

### Constructing a linkage map and the characteristics of markers

A total of 190 DH lines were used for genotyping with MaizeSNP3K chip, which was carried out on the Illumina Golden-Gate SNP genotyping platform [31] and 2956 high-quality SNPs were detected. The missing rate for these SNPs ranged from 0 to 20.00 % (average 1.50 %), the heterozygosity ranged from 0 to 14.21 % (average 2.06 %). A total of 4.74 % (9/190) of the DH lines with SNP heterozygosity ≥ 10 % were excluded in further analysis. Minor allele frequency (MAF) for these SNPs ranged from 0 to 0.50 (average 0.42) (Additional file 1: Table S2). Of these high-quality SNPs, 1318 SNPs were polymorphic between the two parental lines, and the marker distribution frequency for the two parents ranged from 30 to 65 % (Additional file 1: Figure S3). After quality control, 1137 SNPs were left and used to construct a linkage map using the Joinmap4.0 instructions [32]. The total length of the linkage map was 1426.83 cM with an average interval of 1.26 cM (Additional file 1: Table S3).

### QTL characteristics in the DH and haploid populations

**Fig. 3:**
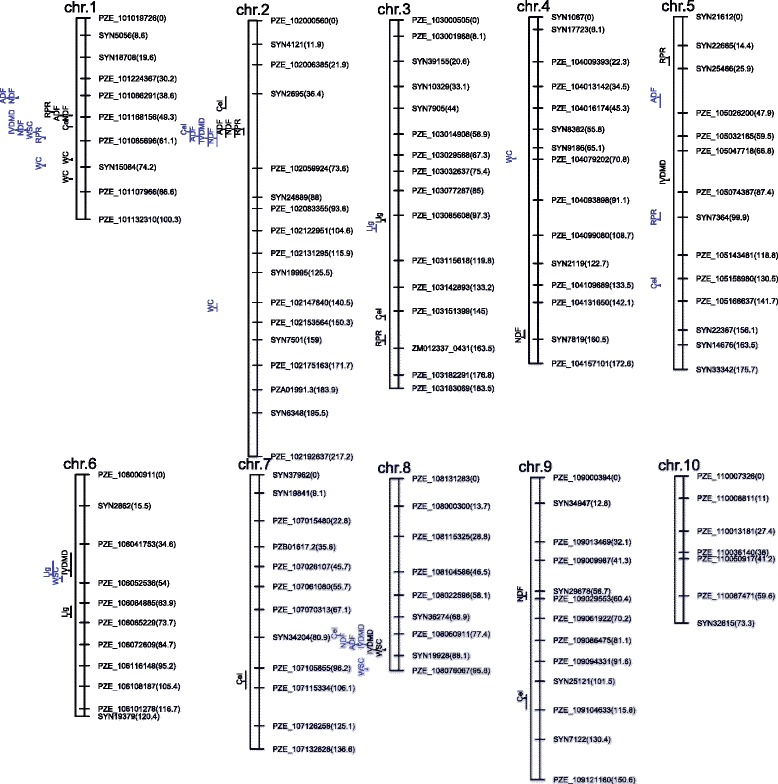
Genetic maps and distribution of putative RPR, IVDMD and other stalk traits QTL in DH and haploid populations. *Blue letters* represented QTL detected in DH population. *Black letters* represented QTL detected in haploid population

Across five environments, the number and position of QTL detected in the DH and haploid populations was shown in Fig. 3. For each trait evaluated in this study, one or more QTL were identified in one region or even shared the same support intervals with the distance of less than 20 cM between the DH and haploid populations.

In the haploid population, four QTL for RPR were detected on chromosomes 1, 2, 3 and 5, two of which were identified on chromosomes 1 and 5 using the DH population (Table 4 and Fig. 3). The position of QTL identified in the haploid population on chromosome 1 was close to that detected in the DH population, which accounted for 6.60 and 8.00 % of the RPR genetic variation, respectively. The favorable alleles of RPR QTL were contributed by the C7-2 parental line in the DH population. All QTL of RPR detected in the DH and haploid populations together explained 25.90 and 42.90 % of the RPR genetic variation, respectively. The favorable alleles of RPR QTL on chromosomes 1, 2 and 3 were contributed by the RPR-higher parent C7-2, while the RPR-lower parent Z58 donated the alleles on chromosomes 1 and 5.

**Table 4:** QTL detected for RPR, IVDMD in DH and haploid populations, respectively

Traits^a^	Bin	Position(cM)^b^	Support interval (cM)	Flanking markers ^c^		LOD	A^d^	*P*_*G*_ (%)
RPR^haploid^	1.07	46.80	46.59–47.06	PZE_101169244	PZE_101164570	3.04	−1.41	6.60
RPR^DH^	1.10	59.40	59.39–60.29	PZE_101080378	PZE_101085696	3.5	1.56	9.00
RPR^haploid^	2.02	54.20	53.64–56.99	PZE_102030524	PZE_102039914	7.09	2.15	15.70
RPR^haploid^	3.09	160.00	157–161.64	PZE_103165581	ZM012337_0431	4.78	1.75	10.30
RPR^haploid^	5.01	20.40	19.83–24.26	PZA01570.1	SYN25466	5.02	−1.76	10.30
RPR^DH^	5.05	101.00	97.79–101.52	PZA00987.1	SYN31361	6.32	−2.18	16.90
IVDMD^DH^	1.04/1.07	55.90	55.29–56.42	PZE_101179982	SYN3987	8.4	3.35	18.50
IVDMD^DH^	2.02/2.03	58.70	53.64–62.59	PZE_102030524	PZE_102039914	3.91	−1.98	8.60
IVDMD^haploid^	5.04	81.50	80.92–81.57	PZE_105099416	PZE_105077111	3.14	1.26	6.80
IVDMD^haploid^	6.03	47.80	39.16–50.73	PZE_106041753	PZE_106051230	3.69	−1.45	8.00
IVDMD^DH^	8.04	82.70	82.42–83.23	PZE_108068741	PZE_108069579	6.49	2.48	16.00
IVDMD^haploid^	8.05	85.40	84.79–85.70	PZE_108069355	PZE_108074750	8.09	2.08	18.60

^a DH^ QTL detected in DH population, ^haploid^ QTL detected in Haploid population

^b^ The peak position with the highest LOD of each QTL

^c^ The Flanking markers of the identified QTL according to B73 reference sequence Version 5.60

^d^ Estimate of allele effect

QTL shown in one frame represented that the genetic distance between them was less than 20 cM

For IVDMD, three QTL were identified in each population, which explained 8.60–18.50 % of total genetic variation in the DH population and 6.80–18.60 % in haploid population These QTL were detected on chromosomes 1, 2, and 8 in the DH population and on chromosomes 5, 6 and 8 in the haploid population. Two QTL detected on chromosome 8 were tightly linked, which explained the 16.00 and 18.60 % of IVDMD genetic variation, respectively, and both were contributed by the IVDMD-higher parent Z58 in the DH and haploid populations.

In the DH population, QTL of RPR, IVDMD, ADF, NDF and WSC shared the same region ranging from 39.91 cM to 59.43 cM on chromosome 1. The QTL intervals for RPR, ADF, NDF and Cel detected in the haploid population ranging from 46.82 cM to 54.19 cM were completely included in the region described above. On chromosome 2, QTL of IVDMD detected in the DH population and the QTL of RPR detected in the haploid population were located adjacent to each other and shared common regions with the QTL of ADF, NDF and Cel. Two or more QTL located in bin 8.04/8.05 for IVDMD, ADF, NDF, Cel, and WSC clustered in the same chromosome region ranging from 78.11 cM to 94.79 cM in the DH and haploid populations.

### Candidate gene identification for RPR and IVDMD in the DH and haploid populations

With a relatively high mapping resolution, some QTL representing the small genomic regions and the linear B73 genome can be used for searching candidate genes related to RPR and IVDMD. Based on the available annotation of the B73 reference sequence Version 5b.60 (http://ftp.maizesequence.org/release-5b/filtered-set/), we applied the MapMan BIN classification [33] and maizeGDB website (http://www.maizegdb.org/) to search for candidate genes.

In this study, four QTL which equally assigned for RPR and IVDMD in the two populations could account for more than 15.00 % of the genetic variation. The number of genes located in the four QTL of RPR and IVDMD were different from each other based on gene screening using qteller3 (http://qteller.com/qteller3/index.php) (Additional file 1: Table S5–S8). Nineteen genes have previously been demonstrated to be associated with cell wall formation mainly involved with cellulose and lignin synthesis (Table 5) [34–43]. Candidate genes participating same bioprocess were predicated between the DH and haploid populations for RPR and IVDMD, which were consistent with the results proposed by previous studies [20, 21, 44]. Moreover, although some evidence illustrated that some transcription factors, such as *NAC*, *R2R3-MYB*, *C2H2, C3HC4 transcription factors families* and so on, were associated with the cell wall [45–52], there was no clear evidence and further investigation was necessary to confirm the function of other annotated genes encoding similar transcription factors.

**Table 5:** Putative candidate genes for RPR and IVDMD in two different ploidy populations

Traits	Population ploidy	Bin	Interval (Mb)	Putative candidate gene Id	References	Biological pathway
RPR	DH	5.05	176–178	GRMZM2G132706	[34, 35]	cellulose biosynthesis
RPR	haploid	2.02	13–20	GRMZM2G044884	[34, 35]	cellulose biosynthesis
				GRMZM2G120016		
				GRMZM2G168474		
				GRMZM2G162333	[36]	biodegradation pathway of the cell wall
				GRMZM2G114276	[38]	affecting cell wall composition
				GRMZM2G045398	[37]	controlling the expression of cellulose synthase genes
				GRMZM2G318408		
				GRMZM2G476597	[39]	lignin biosynthesis
				GRMZM2G020500		
IVDMD	DH	8.04	120–121	GRMZM2G042865	[34, 35]	cellulose biosynthesis
				GRMZM2G074631		
				GRMZM2G179444	[36]	biodegradation pathway of the cell wall
IVDMD	haploid	8.05	121–129	GRMZM2G467497	[40]	depositing lignin in the cell wall
				GRMZM2G381129		
				AC209819.3_FG005	[43]	lignin biosynthesis
				AC205471.4_FG008	[41]	affecting cell wall composition
				GRMZM2G117198	[42]	lignin biosynthesis
				GRMZM2G071339	[36]	biodegradation pathway of the cell wall

### Transcriptional expression analyses of key genes involved in lignin and cellulose synthesis for haploid and diploid version of parental lines

**Fig. 4:**
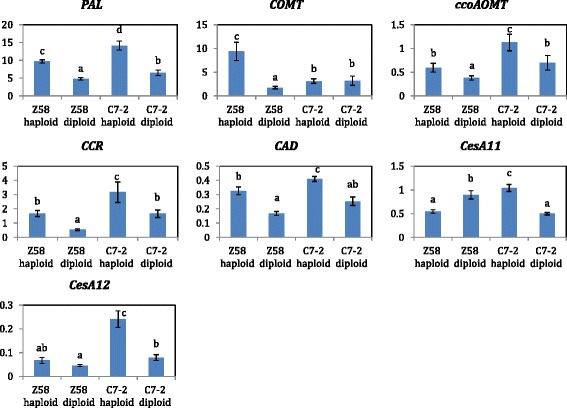
Expression levels of lignin and cellulose synthesis genes in FIAG rind of haploid and diploid parental lines at milky stage. Quantitative RT-PCR analysis for lignin and cellulose synthesis genes was shown in the first five pictures and the last two pictures, respectively (*ACTIN* as an internal control). Four bars in each picture presented Z58 haploid, Z58 diploid, C7-2 haploid and C7-2 diploid from left to right. Different lowercase indicated that statistical significant difference (*P* < 0.05). Error bar ± SD

To determine whether key genes involved in lignin biosynthesis were ploidy-modulated at a transcriptional level, relative expression levels of five genes, *PAL, COMT, ccoAOMT, CCR* and *CAD*, were analyzed (Fig. 4). In parental line Z58, transcript levels of the five genes increased 1.57–5.30folds in haploid plant relative to diploid plants. Particularly, *COMT* had the largest change fromZ58 in diploids to Z58 in haploids, while with no significant change from C7-2 in diploids to C7-2 in haploids. In the other parental line C7-2, higher expression levels of haploids than diploids were also observed across five genes, however, the changes of expression level (1.61–2.12-fold) from diploids to haploids were lower than what observed in Z58.

We also examined expression of cellulose synthesis genes encoding glycosyltransferase which detected in both populations for RPR and IVDMD (Fig. 4), and *CesA11* and *CesA12* were consistently co-expressed at all developmental stages of and were predominantly associated with the deposition of the secondary cell wall in maize stems even after the anthesis stage [53]. For C7-2, the two genes, *CesA11* and *CesA12*, were up-regulated 2.08–fold and 3.04–fold, respectively, in the haploid version relative to the diploid version. For Z58, the expression of *CesA12* increased 1.48 fold in haploids in comparison to diploids, however, haploids had lower expression level for *CesA11*.

## Discussion

### Performance and heritability of stalk traits in DH and haploid populations

The previous genetic investigations on RPR and IVDMD in diploid populations revealed that the traits were likely polygenic in maize and were affected by several mechanisms and complicated by confounding factors.

In this study, two parent lines presented consistent trends on RPR, as well as other traits, between the DH and haploid populations. This result showed each parent contributed coherent negative or positive allele effects even under different genome dosages and additive effect may played important role for partial phenotypic variation.

Heritability estimation depended on genetic background of the material, population types surveyed, interaction with environments and experimental design [54]. In this study, the heritability of RPR was 0.72 and 0.78 estimated in DH and haploid population respectively, which were all in agreement with a high heritability of RPR reported in previous studies [19–21]. However, Peiffer et al. [22] reported that the heritability of RPR estimated from 26 RIL families of maize nested association mapping (NAM) population ranged from 0.08 to 0.34 (average 0.21), which may be due to a wide range in the flowering time among NAM families. Likewise, a high heritability was obtained for IVDMD in both DH (0.81) and haploid (0.89) populations, which were different from a moderate heritability, ranging from 0.55 to 0.68, reported in previous studies [55–58]. Moderate or high heritability values were obtained for other stalk traits as well. In conclusion, the high heritability values of stalk traits evaluated in this study could provide solid basis for QTL mapping analysis.

For most of traits evaluated in this study, the heritability estimated from the haploid population was higher than that from the DH population. Our results were consistent with heritability of DH and its haploid populations in maize reported by Geiger et al. [16], although different traits were studied. The higher heritability estimated from the haploid population can be explained by the relatively smaller *σ*_*G* × *E*_^2^ and *σ*_*e*_^2^ in haploid population than in DH population, which can be explained by that haploid lines mainly reacted more sensitively than DH lines to biotic and abiotic stress and therefore effectively uncover susceptibility to diseases and outer constraints, which had been proposed by Chase et al. [59] and Geiger et al. [16]. In addition, all traits evaluated in this study were measured with high precision and then had a solid genetic basis in two ploidy populations, fundamentally suggesting that the haploid population as well as the DH population could be used in QTL analysis.

### Phenotypic correlations and QTL co-localization for the same trait between the DH and haploid populations

Geiger et al. [16] reported moderate to high correlations between the DH and haploid lines from three material sets (KWS, SWS, and MON) for early vigor, silking, plant height, and stover weight per plant. We also observed a significant (*P* < 0.0001) moderate to strong positive correlation (*r* = 0.38-0.69) between the DH and haploid populations for all stalk traits (Fig. 2). This could suggest that moderate to strong correlations can occur independently of material background and trait restrictions. This high correlation between haploids and corresponding DH lines may provide reference information for maize breeders to select desirable lines at haploid stage, which could reduce breeding costs. However, the genetic mechanism on the connection between the DH and haploid populations has not yet been studied and therefore, is still unclear. In this study, through QTL mapping studies conducted in DH and its haploid population, we intended to understand this issue in term of genetic architecture. We first identified common QTL regions between the DH and haploid populations for each stalk trait, which could be considered as the genetic reason for the phenotypic correlation. Other QTL located on different chromosomes or having larger distance (>20 cM) may be partially caused by the change of genome dosage and explained by the different population size. Ming et al. [60] reported that many QTL for sugar content detected in sugarcane autopolyploids were not consistent with known candidate genes and suggested that other approaches will be necessary to isolate the genetic determinants of high sugar content of vegetative tissues. Until now, QTL detection in haploid population has not been reported.

### Phenotypic correlations and QTL co-localizations among different traits

In an attempt to further understand the genetic architecture of RPR and IVDMD in maize, genomic regions for RPR, IVDMD and other stalk component traits were compared and phenotypic correlations between RPR, IVDMD and other stalk component were evaluated. Forty-seven QTL were identified in the DH and haploid populations (Additional file 1: Table S4, Fig. 3 and Table 4). The incidence of QTL clusters in similar genomic regions reflected trait associations [61].

Two studies have proposed that genes associated with the biosynthesis of cell wall components were considered as candidate genes for RPR [19, 20]. We also observed the positive correlations and QTL co-location of RPR with ADF, NDF and Cel, which were consistent with previous studies. RPR was negatively correlated with WC in a high-oil RIL population [20]. The same correlation trend of RPR with WC and WSC were observed in the DH and haploid populations, except that RPR had no correlation with WC in the haploid population. In addition, Hu et al. [20] reported that the internode diameter, fresh weight of internode and dry weight of internode were also significantly positively correlated with RPR, and the difference in planting years, densities and maize varieties led to different stalk RPRs [62].

IVDMD showed the opposite correlation direction as the correlations of RPR with ADF, NDF, Cel and WSC, and had the same correlation direction as the correlations of RPR with WC. Therefore, WC may be one of the improved elements for practical breeding for stalk lodging resistance and forage maize. Several QTL associated with IVDMD and other stalk components were located in the same bins as identified in our studied [57, 58, 63]. Lig was positively correlated with IVDMD and was not correlated with RPR, which was not in agreement with previous studies [20]. This may be due to the no-forage background materials used in this study. No reports were available on QTL both for RPR and IVDMD. Only in the DH population evaluated in this study, we first detected one QTL cluster for RPR and IVDMD at bin 1.10 and bin 1.07, respectively. However, we found more than one QTL of RPR or IVDMD sharing common regions or flanking markers with the QTL of other stalk components, which suggested close linkage or pleiotropy as the explanation for the correlations and some common genes had effects on RPR and IVDMD. The QTL clusters could be deployed for improving RPR and IVDMD in maize through marker-assisted selection.

### Compare QTL identified in this study with those identified in previous studies in diploid populations

We have identified additive QTL for RPR on chromosomes 1, 2, 3 and 5. Flint-Garcia et al. [19] detected one QTL region on chromosome 3 contained overlapping support intervals across four F2:3 maize populations. Also, in less than four populations, other QTL were detected at bins 1.07–1.09, 2.02, 2.06–2.07, 3.04–3.08, and 5.02. Similarly, our mapping study of the DH and haploid populations identified five RPR QTL located near bins 1.07, 1.10, 2.02, 3.09 and 5.01. Hu et al. [20] investigated RPR in a RIL population derived from a high-oil population and reported that RPR QTL were detected on all chromosomes except for chromosome 5 and the QTL located in bin 3.06 was the most important one and it accounted for 12 % of the phenotypic variation. Li et al. [21] identified seven RPR-associated QTL in two RIL populations. Among these QTL, the largest-effect QTL accounted for 18.9 % of the phenotypic variation was located at bin 3.06, and other QTL for RPR were observed at bins 2.10, 3.08, 9.03–9.04, 4.06, 6.05, and 6.07, explaining 4.40–13.80 % of the phenotypic variation. In the present study, the QTL location on chromosome 3 were only detected in the haploid populations and accounted for 10.30 % of the genetic variation with highly detected frequency in 1000 runs cross-validation (Additional file 1: Figure S4). Moreover, it is worth noting that RPR-associated QTL, which were observed at bin 2.02 in the haploid populations and explained more than 15.00 % of the contribution to genetic variation, were located in the same region as QTL detected by Flint-Garcia et al. [19]. The QTL detected at bin 5.05 in the DH population could account for the highest percentage of RPR genetic variation (up to 16.90 %) and were not located in the QTL cluster with other traits, and this QTL has not been proposed by previous studies. Since these two newly discovered QTL were also detected with high frequencies in the 1000 cross-validation, this confirmed our conclusion that QTL at bins 2.02 and 5.05 likely carried major candidate genes for RPR (Additional file 1: Figure S4).

Six QTL for IVDMD in total were detected in DH and its haploid population in this study. Two QTL detected in the DH and haploid populations were located in adjacent bins 8.04 and 8.05 with a genetic distance of less than 3 cM. These two QTL also showed high detection frequencies in cross-validation (Additional file 1: Figure S4). Similarly, Wei et al. [57] reported that a IVDMD QTL located at bin 8.06–8.07 were detected in Pop2 combined analysis, which was adjacent to QTL for IVDMD on chromosome 8 detected in this study. Other QTL for IVDMD identified in the DH and haploid populations were distributed on chromosomes 1, 2, 5 and 6. IVDMD QTL located at bin 1.07 can explain 18.50 % of the genetic variation. Previous reports showed that QTL on chromosome 1 had a great effect on stalk digestibility [57, 63]. The QTL located at bins 5.02–5.03 and 5.03–5.06 were detected in Xuchang and Luoyang Pop2, respectively, by Wei et al. [57]. Wang et al. [58] suggested that IVDMD QTL explained more than 10 % of the genetic variation in both F3 and F4 generations were mapped on the same genomic position on chromosome 6, which were the same as QTL detected in maize recombinant inbred line progeny of F288 × F271 [64]. One IVDMD QTL detected in our study was also on chromosome 6. These QTL described above were closely linked under high-density SNP markers and deserve further investigation for finding candidate genes underlying IVDMD in a no-forage genetic background.

### The role of genome dosage changes on gene expression of lignin and cellulose synthesis in inbred and haploids of two parental lines

Most candidate genes were involved in lignin and cellulose synthesis which affect the stalk cell wall structure. Lignin was a phenolic polymer that imparted mechanical strength of the plant secondary cell wall, and therefore, was considered to confer stalk rot resistance and involve in plant evolution [65]. Particularly, genes participating in lignin synthesis were identified only in haploid population in our study. Therefore, based on the gene function annotations for RPR and IVDMD QTL detected in the DH and haploid populations, we analyzed the key gene expressions of lignin and cellulose synthesis and genome dosage regulation. The expression levels and phenotypes showed several interesting results, suggesting a partial explanation for ploidy effect mechanisms in the haploid condition.

In the one dosage genome, compared to the inbred, *CesA11 and CesA112* gene expressions were up-regulated except for the *CesA11* and *CesA12* gene in Z58, which was consistent with the decreased Cel content in haploid Z58 and higher content in C7-2 haploids (Fig. 4). Unlike other gene expressions in lignin synthesis, the *COMT* gene showed significantly lower expression levels in C7-2 haploids than that in Z58 haploids. All these results illustrated the existence of genetic variation in morphological responses to ploidy changes, which was consistent with the results which reported by Riddle et al. [66].

All lignin and synthesis gene expression showed dramatically inversed expression levels, except for *CesA11* in Z58, as genome dosage number increased (Fig. 4), which was consistent with higher Lig contents for two parents and increased Cel contents for parent line C7-2 in the haploid condition and can be explained by dosage compensation [67]. These findings implied that the major effect of genome dosage changes were not simply proportional to copy number of regulatory genes and may not be directly related to the phenotype, but rather result from various regulatory components which resulted in compensation for gene expression in the one dosage genome. Previous studies had also reported that many target loci exhibited dosage compensation, such as *Adh* expression with reduced alleles number [68]. Quantitative traits trended to be regulated by genes exhibiting dosage effects and most were transcription factors [69, 70]. Forty-seven dosage-dependent modifiers operated in macromolecular complexes in a regulatory network [71]. Many other mechanisms, such as siRNA, DNA methylation and so on, may be concerned with expression changes in dosage- sensitive region genes, which can also sharpen the evolution of copy-number varied regions [72–74].

## Conclusions

Using DH and its corresponding haploid populations, this analyse revealed important genomic regions associated with eight stalk-related traits. These QTL explained a large extent of phenotypic variance. One or more QTL sharing common region for each stalk-related trait were detected between this two different ploidy populations. The heritabilities in haploid population were higher than DH population in all stalk traits except WC. This study identified candidate genes involving in lignin and cellulose synthesis for RPR and IVDMD, which were the two most important stalk traits. The expression levels of most of these candidate genes were significant higher in haploid parents than that in corresponding diploid parents. Haploid population may be used as one of platforms providing information on the genetic basis for stalk-related traits, and the genetic connection between DH and haploids for traits can facilitate the selection of materials at haploid-stage which can boost the practical maize breeding.

The dosage compensation mechanism and dosage-sensitivity genes may be further examined by analyzing the genetic architecture of certain traits by comparing QTL mapping results between different ploidy populations, which may also have important implications for understanding gene regulatory networks and genome evolution.

## Methods

### Materials and population construction

The parental lines of two populations belong to two distinct maize germplasm groups. The maternal inbred line Zheng58 (Z58) is dent corn of the Reid heterotic group, and the paternal inbred line Chang7-2 (C7-2) is flint corn. The single cross (ZD958) of these two parental lines is one of the most widely planted commercial varieties in China.

The DH population was constructed by production of haploids with an in vivo haploid induction procedure and followed by chromosome doubling (Additional file 1: Figure S1).

In total, the DH population consisted of 190 DH lines. Accordingly, the haploid population was constructed by crossing each DH line, used as source germplasm, with a haploid inducer, used as pollinator, and we finally produced 170 haploid lines. To obtain enough DH and haploid seeds for evaluating phenotypes in field trial, in the experimental station of the China Agricultural University (Yacheng in Hainan Province), we planted two replications of 190 DH lines with 21 plants in each plot that again pollinated with inducer line CAU5 at the anthesis stage [75]. The haploid seeds were manually picked up from the harvested ears based on the R1-nj color markers [76].

### Field experiments

In 2013 and 2014, the two populations, their parental lines (inbred and haploid) and F_1_ generation were sown in two experimental stations of the China Agricultural University (Shangzhuang in Beijing and Quzhou in Hebei Province), and Shijiazhuang experimental station of Hebei Academy of Agriculture and Forestry Sciences. At each location, the two populations were adjacently planted to reduce the influence of field heterogeneity. We used a randomized complete block design with two blocks for both populations. Within each block, each line was assigned to a single row plot (Additional file 1: Figure S2).

### Trait evaluations

The fourth internode above ground (FIAG) was measured for nine traits described as follows. Three randomly selected plants in each row (i.e., plot) were chosen for trait evaluation. RPR was measured with an electronic penetrometer (AWOS-SL04, Aiwoshi Company, Hebei, China) at the milky stage. The measurement of RPR and water content (WC) of the internode, as well as measurement of IVDMD, acid detergent fiber (ADF), neutral detergent fiber (NDF), lignin (Lig), cellulose (Cel) and water soluble carbohydrate (WSC) followed a standard procedure described by Hu et al. [20].

### Phenotypic data analysis

In order to evaluate phenotypic traits, all DH lines and haploid lines, were planted in 3 locations in 2013 and 2014, which were treated totally as 5 independent macro environments (not including Quzhou 2014 due to large proportion of missing data points,). Then a linear model was used to perform an analysis of variance (ANOVA), genotypic value estimation (BLUE) and variance components estimation for the DH and haploid populations for each trait:

$$ Y=\mu +G+E+G\times E+R(E)+\varepsilon $$

where *μ* is the grand mean, *G* is genotypic effect, *E* is the environment effect, *G* × *E* is the genotype-by-environment interaction, *R*(*E*) is the effect of block within environment, and *ε* is the random error. All statistical analyses were performed using SAS version 9.3 [77]. The genotypic effect *G* was estimated when it was treated as a fixed effect using LSMEANs with PROC GLM. The genotypic value estimates (BLUEs) were used to calculate correlation coefficients by PROC CORR with Pearson’s method. The variance components were estimated using PROC VARCOMP with the method of restricted maximum likelihood (REML). The estimates of genotypic variance (*σ*_*G*_^2^), genotype-by-environment interaction (*σ*_*G* × *E*_^2^) and random error (*σ*_*e*_^2^) were used to estimate heritability based on the formula [78]:

$$ {h}_B^2=\frac{\sigma_G^2}{\sigma_G^2+\frac{\sigma_{G\times E}^2}{l}+\frac{\sigma_e^2}{rl}} $$

where *l* is the number of macro environments and *r* is the number of blocks within each environment, which equals 5 and 3, respectively in the study.

### Genotyping and genetic map construction

Genomic DNA was extracted from young leaves of DH and the parental lines using a CTAB method [79], and was then purified. The purified DNA was genotyped with the maizeSNP3K chip (3,072SNPs), which is a subset of the Illumina MaizeSNP50 BeadChip [80]. SNP genotyping was performed on the Illumina Golden-Gate SNP genotyping platform at the National Maize Improvement Center of China of the China Agricultural University. Checking the quantity of each SNP was carried out manually as described by Yang et al. [81]. The qualified SNPs were reserved for further screening and creation of linkage maps.

After genotyped, 1228 polymorphic markers between the two parental lines were determined. Subsequently, heterozygosity of each line, the missing rate, minor allele frequency (MAF) and heterozygosity of each SNP were calculated. The DH lines with heterozygosity ≤ 0.1 and the SNPs having polymorphisms between two parents with a missing rate ≤ 0.2 and MAF ≥ 0.05 were selected to construct a genetic linkage map with software package Joinmap4.0. SNPs and lines that shared 100 % similarities were deleted according to the Joinmap4.0 instructions [32]. The linkage groups were composed at a minimum Logarithm of odds (LOD) of 7, and the Kosambi mapping function and regression-mapping algorithm were used for calculating map distances.

### QTL analysis

Since the segregating populations used in this study were DH and haploid populations, an additive genetic model was chosen for QTL analysis, using the BLUEs across environments as phenotypic data. Composite interval mapping (CIM) with a regression approach [82] in combination with the use of cofactors was employed [83]. A two-step procedure was utilized for QTL detection as described by Hu et al. [84]. A threshold of LOD =3.0 was used to determine QTL for all traits based on 2000 permutations [85]. 1-LOD support intervals were calculated from the significant peak to a certain position on both sides along the chromosome at which the LOD score had a 1.0 unit decrease [86]. To facilitate comparisons among linked QTL, two QTL were designated as overlapping when they were separated by less than 20 cM [87]. The total proportion of genotypic variation (*p*_*G*_) explained by the detected QTL was calculated by the formula *p*_*G*_ = *R*_*adj*_^2^/*h*_*B*_^2^ [78]. Fivefold cross-validation was used to assess the reliability of QTL mapping results with 1000 runs [88].

Physical positions of QTL were obtained from the physical positions of their flanking markers. All genes between the flanking markers of each QTL were extracted from the filtered gene set of the maize genome sequence (http://ftp.maizesequence.org/release-5b/filtered-set/) by qteller3 (http://qteller.com/qteller3/index.php), assuming a linear relationship between recombination and physical distances within this interval.

### RNA extraction and RT-PCR

Stalk rind used in gene expression analysis was collected from plant FIAG at the milky stage. Total RNA was extracted using Ultrapure RNA Reagent (Cat#CW0581, CWbio.Co.Ltd, Beijing, China) according to the manufacturer’s instructions. Total RNA was digested with RNase-Free DNase I to remove any DNA (Cat# CW2090, CWbio.Co.Ltd, Beijing, China). cDNA was synthesized using HiFi-MMLVcDNA (Cat#CW0744, CWbio.Co.Ltd, Beijing, China). Two microliters of diluted cDNA was used for quantitative RT-PCR analysis using the primer pairs listed in Additional file 1: Table S1. An ABI7500 machine was used to conduct RT-PCR analysis [89]. *Actin* was used as an internal control to calculate the relative expression levels in three biological replications using the analysis method of 2-^△△CT^ [90].

### Availability of supporting data and materials

The data supporting the results of this article can be found in the article and its additional files. The biological sequences used for gene RT-PCR in this study come from National Center for Biotechnology Information (NCBI) under the accession numbers L77912, M73235, AJ242981, NM_001112018, AJ005702, AY372245, AY372246 and DQ492681. All published datasets referred to in the manuscript are cited in the reference list.

## Additional file

Additional file 1: Figure S1. Route used to develop DH population and the corresponding haploid populations. **Figure S2.** Presentation of DH and haploid populations planted in field. **Figure S3.** Histogram for genotypic percentage in DH population. **Figure S4.** QTL detected frequency for RPR and IVDMD in DH and haploid populations with five-split cross validation for 1000 times. For RPR, the QTL at bin 2.02 and 5.05 were detected in DH and haploid population with high frequencies in the 1000 cross-validation, respectively. For IVDMD, the QTL at bin 8.04 and 8.05 were detected in DH and haploid population with high frequencies in the 1000 cross-validation, respectively. **Table S1.** Primer sequences of genes involved in lignin and cellulose synthesis for RT-PCR. **Table S2.** Summary of SNP characteristics of DH population. **Table S3.** Summary of the linkage map characteristics DH population. **Table S4.** QTL detected for other stalk traits in DH and haploid populations. **Table S5.** Annotation of the 45 predicted genes located within putative RPR QTL interval in DH population. **Table S6.** Annotation of the 191 predicted genes located within putative RPR QTL interval in haploid population. **Table S7.** Annotation of the 24 predicted genes located within the putative IVDMD QTL interval in DH population. **Table S8.** Annotation of the 191 predicted genes located within putative IVDMD QTL interval in haploid population. (DOCX 870 kb)

## Abbreviations

ADF: Acid detergent fiber
BLUE: best linear unbiased estimation
C7-2: Chang7-2
*CAD*: Cinnamyl alcohol dehydrogenase gene
*ccoAOMT*: Caffeoyl CoA O-methyltransferase gene
*CCR*: Cinnamoyl CoA reductase1 gene
Cel: cellulose
*CesA11*: cellulose synthase catalytic subunit 11
*CesA12*: cellulose synthase catalytic subunit 12
CIM: composite interval mapping
cM: centimorgan
*COMT*: O-methyltransferase gene
CV: coefficients of variation
DH: doubled haploid
FIAG: fourth internode above ground
IVDMD: in vitro dry matter digestion
Lig: lignin
MAF: minor allele frequency
NDF: neutral detergent fiber
*PAL*: phenylalanine ammonia-lyase gene
*p*_*G*_: proportion of genotypic variation
QTL: quantitative trait loci
RPR: rind penetrometer resistance
SNP: single nucleotide polymorphism
WSC: water soluble carbohydrate
Z58: Zheng58
