# Supplementary material for: Ploidy effect and genetic architecture exploration of stalk traits using DH and its corresponding haploid populations in maize
Source: BMC Plant Biol. 2016 Feb 25;16:50. doi: 10.1186/s12870-016-0742-3 (PMC4766647; doi:10.1186/s12870-016-0742-3)
Supplement: Additional file 1: Figure S1. — Route used to develop DH population and the corresponding haploid populations. Figure S2. Presentation of DH and haploid populations planted in field. Figure S3. Histogram for genotypic percentage in DH population. Figure S4. QTL detected frequency for RPR and IVDMD in DH and haploid populations with five-split cross validation for 1000 times. For RPR, the QTL at bin 2.02 and 5.05 were detected in DH and haploid population with high frequencies in the 1000 cross-validation, respectively. For IVDMD, the QTL at bin 8.04 and 8.05 were detected in DH and haploid population with high frequencies in the 1000 cross-validation, respectively. Table S1. Primer sequences of genes involved in lignin and cellulose synthesis for RT-PCR. Table S2. Summary of SNP characteristics of DH population. Table S3. Summary of the linkage map characteristics DH population. Table S4. QTL detected for other stalk traits in DH and haploid populations. Table S5. Annotation of the 45 predicted genes located within putative RPR QTL interval in DH population. Table S6. Annotation of the 191 predicted genes located within putative RPR QTL interval in haploid population. Table S7. Annotation of the 24 predicted genes located within the putative IVDMD QTL interval in DH population. Table S8. Annotation of the 191 predicted genes located within putative IVDMD QTL interval in haploid population. (DOCX 870 kb) [file 12870_2016_742_MOESM1_ESM.docx]

**Additional file 1**

Supplemental information contains 4 Supplemental Figures and 8 Supplemental Tables

**Supplementary Figures**

**ZD958 (F1)**

**Picking up haploids identified by color**

**Selfing**

**Induced by Cau5**

**Doubling and selfing**

.........

.........

.........

**Induced by CAU5**

**Figure S1 Route used to develop DH population and the corresponding haploidpopulations.**


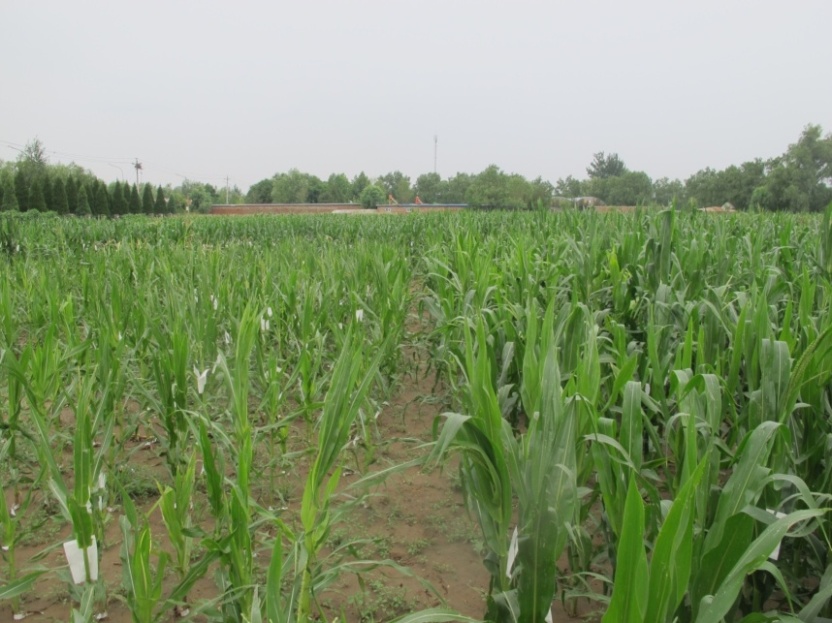

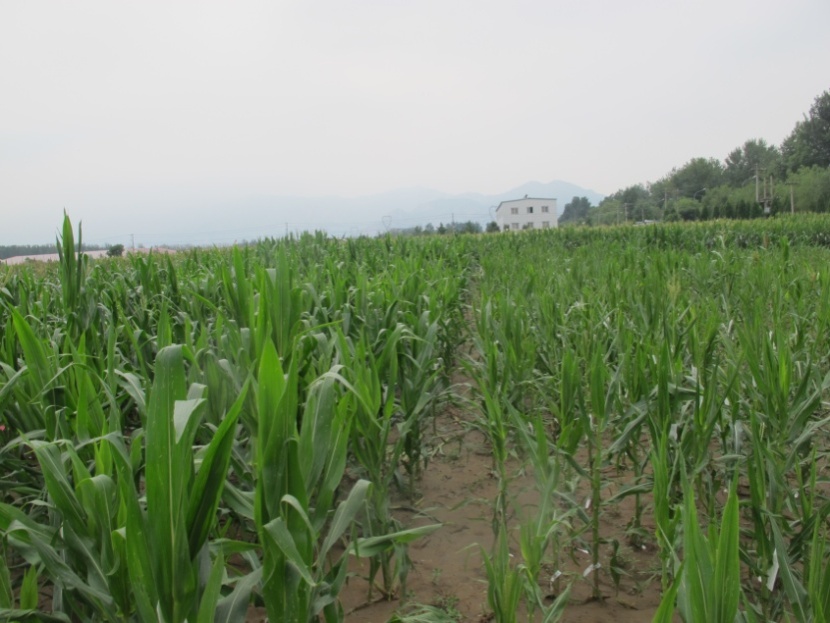


**DH population**

**DH population**

**haploid population**

**haploid population**

**Figure S2 Presentation of DH and haploid populations planted in field.**


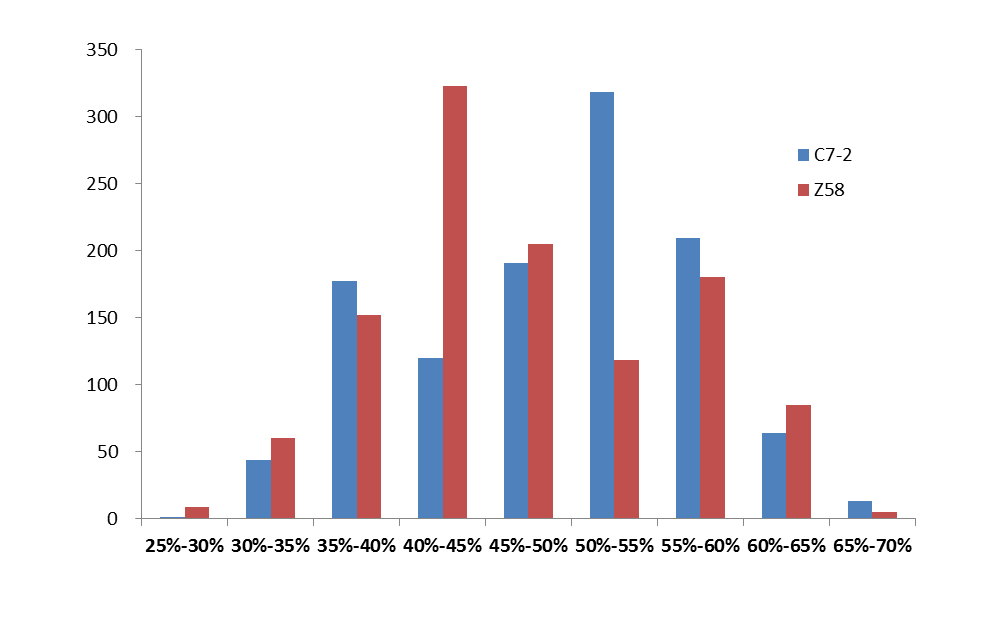


**Number of SNP markers**

**Figure S3 Histogram for genotypic percentage in DH population.**

**
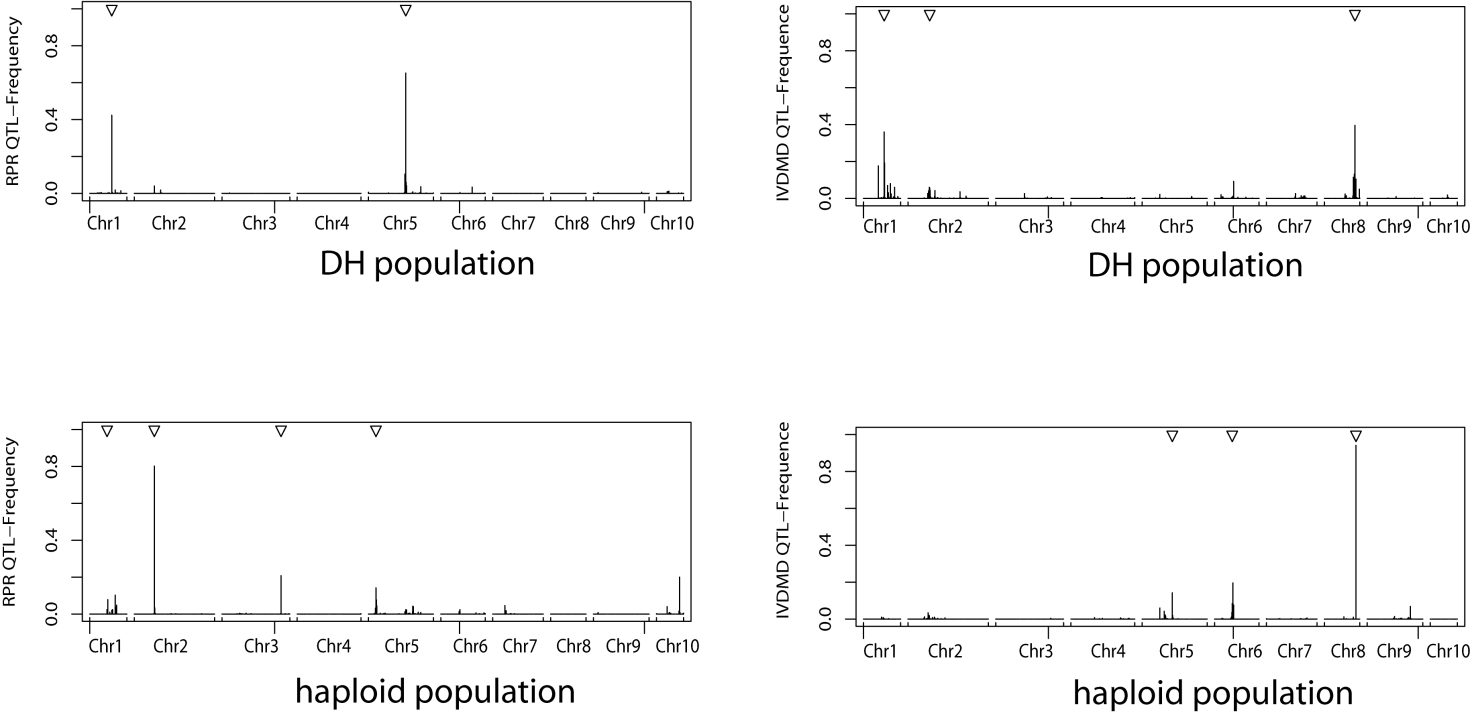
**

**Figure S4 QTL detectedfrequency for RPR and IVDMD in DH and haploid populations with five-split cross validation for 1000 times.**

For RPR, the QTL at bin 2.02 and 5.05 were detected in DH and haploidpopulation with high frequencies in the 1,000 cross-validation, respectively. For IVDMD, the QTL at bin 8.04 and 8.05 were detected in DH and haploid population with high frequencies in the 1,000 cross-validation, respectively.

**Supplemental Tables**

**Table S1 Primer sequences of genes involved in lignin and cellulose synthesis for RT-PCR.**

| Name | Accession numbers | Forward primer | Reverse primer |
| --- | --- | --- | --- |
| *PAL* | L77912 | CACGGACCACCTGACCCACAA | GCGTACCTGTCCTGCTTGGGC |
| *COMT* | M73235 | ACTCTTGCCCTGTCTGTTCCTTGTA | CACCGCGTCCTTGAGATAGTACCT |
| *ccoAOMT* | AJ242981 | ACATCAACCGCGAGAACTACGAGCT | CGCTCGTGGTAGTTGAGGTAGTTGT |
| *CCR* | NM_001112018 | GGCACCGTCAGGAACCCAGAT | AGATGGCGTCGTAGTCCAGCAGA |
| *CAD* | AJ005702 | TCGGGGCTTCAAAGTATCCTAT | TATGACCAGATCTTCTTGTTGCAGT |
| *CesA11* | AY372245 | TGGAGTGGCATCGGGATCG | CACCGTCGTCCACTTGAACAG |
| *CesA12* | AY372246 | ATCTATCGGACCGTCTCAACCAGG | GCCACTTGAGGTTGCCGTTCTT |
| *ACTIN* | DQ492681 | TCACTACGACTGCTGAGCGAGAAAT | AATAGAACCTCAGGGCACCTAAACC |

**Table S2 Summary of SNP characteristics in DH population.**

|  |  | Mean±SD |  | Range | |
| --- | --- | --- | --- | --- | --- |
| High-quality SNP number |  | 2956 |  |  |  |
| MAF(%) |  | 0.42±0.06 |  | 0.00 | 0.50 |
| SNP heterozygosity in each line(%) |  | 2.06±4.24 |  | 0.00 | 25.81 |
| SNP missing rate in each line (%) |  | 1.50±2.51 |  | 0.03 | 15.36 |
| SNP missing rate (%) |  | 1.50±2.47 |  | 0.00 | 20.00 |
| SNP heterozygosity (%) |  | 2.06±1.95 |  | 0.00 | 14.21 |

**Table S3 Summary of the linkage map characteristics DH population.**

| Chromosome |  | Number of markers |  | Length (cM) |  | Minimum interval (cM) |  | Maximum interval (cM) |  | Average interval (cM) |
| --- | --- | --- | --- | --- | --- | --- | --- | --- | --- | --- |
| 1 |  | 144 |  | 100.34 |  | 0.002 |  | 5.80 |  | 0.70 |
| 2 |  | 132 |  | 217.19 |  | 0.001 |  | 19.60 |  | 1.65 |
| 3 |  | 90 |  | 183.46 |  | 0.007 |  | 12.56 |  | 2.04 |
| 4 |  | 171 |  | 172.55 |  | 0.002 |  | 16.13 |  | 1.01 |
| 5 |  | 111 |  | 175.74 |  | 0.001 |  | 16.69 |  | 1.58 |
| 6 |  | 73 |  | 120.41 |  | 0.002 |  | 15.22 |  | 1.65 |
| 7 |  | 89 |  | 136.62 |  | 0.008 |  | 12.76 |  | 1.54 |
| 8 |  | 172 |  | 95.61 |  | 0.001 |  | 7.63 |  | 0.56 |
| 9 |  | 86 |  | 150.59 |  | 0.029 |  | 20.16 |  | 1.75 |
| 10 |  | 69 |  | 74.32 |  | 0.014 |  | 13.59 |  | 1.08 |

**Table S4 QTL detected for other stalk traits in DH and haploid populations.**

| Traits | Bin | Position(cM)^a^ | Support interval (cM) | Flanking markers ^b^ | | LOD | A^c^ | *P_G_* (%) |
| --- | --- | --- | --- | --- | --- | --- | --- | --- |
|  |  |  |  |  |  |  |  |  |
| WC^haploid^ | 1.09 | 70.69 | 70.11-70.98 | PZE_101087901 | PZE_101215138 | 4.67 | -0.79 | 14.70 |
| WC^DH^ | 1.09 | 73.48 | 73.07-73.86 | PZE_101092737 | SYN15084 | 5.79 | -1.06 | 25.50 |
| WC^haploid^ | 1.05 | 80.18 | 80.01-80.37 | PZE_101101518 | PZE_101117162 | 5.6 | -0.88 | 17.80 |
| WC^DH^ | 2.07 | 143.1 | 141.12-144.92 | PZE_102147840 | PZE_102151093 | 3.85 | -0.88 | 14.40 |
| WC^DH^ | 4.06 | 70.59 | 70.39-70.70 | PZE_104075114 | PZE_104079202 | 3.91 | -0.72 | 11.40 |
| ADF^DH^ | 1.07 | 39.91 | 39.28-40.10 | PZE_101066291 | SYN29479 | 5.02 | -1.56 | 11.50 |
| ADF^haploid^ | 1.09 | 48.62 | 48.35-48.67 | PZE_101165770 | PZE_101205714 | 6.39 | -1.71 | 15.90 |
| ADF^haploid^ | 2.02 | 54.19 | 53.64-58.11 | PZE_102030524 | PZE_102039914 | 6.00 | 1.63 | 14.80 |
| ADF^DH^ | 2.02/2.03 | 57.55 | 53.64-61.47 | PZE_102030524 | PZE_102039914 | 5.73 | 1.73 | 12.30 |
| ADF^DH^ | 5.01 | 40.18 | 38.65-45.16 | SYN37537 | PZE_105026200 | 3.73 | -1.34 | 7.40 |
| ADF^DH^ | 8.04 | 82.67 | 82.42-83.23 | PZE_108068741 | PZE_108069615 | 6.12 | -1.79 | 14.90 |
| NDF^DH^ | 1.07 | 39.91 | 39.28-40.10 | PZE_101066291 | SYN29479 | 6.80 | -1.09 | 1.60 |
| NDF^haploid^ | 1.09 | 48.62 | 48.35-48.67 | PZE_101165770 | PZE_101205714 | 6.77 | -2.17 | 16.20 |
| NDF^DH^ | 1.04/1.07 | 55.86 | 55.29-56.42 | PZE_101179982 | SYN3987 | 3.51 | -2.88 | 6.80 |
| NDF^haploid^ | 2.02 | 54.19 | 53.64-58.11 | PZE_102030524 | PZE_102039914 | 4.09 | 1.71 | 10.10 |
| NDF^DH^ | 2.02/2.03 | 58.67 | 53.64-62.59 | PZE_102030524 | PZE_102039914 | 3.65 | 2.18 | 9.90 |
| NDF^haploid^ | 4.10 | 159.53 | 155.99-160.02 | SYN24017 | SYN7819 | 3.13 | 1.48 | 7.30 |
| NDF^DH^ | 8.04 | 81.66 | 81.45-81.92 | PZE_108068136 | PZE_108068741 | 5.03 | -2.37 | 13.60 |
| NDF^haploid^ | 9.02/9.03 | 59.14 | 57.28-60.67 | SYN29878 | PZE_109037900 | 3.69 | -1.6 | 8.40 |
| Lig^haploid^ | 3.05 | 99.62 | 99.24-100.43 | PZE_103089927 | SYN28295 | 3.60 | -0.4 | 11.00 |
| Lig^DH^ | 3.05 | 103.91 | 102.06-105.48 | SYN28295 | PZE_103098628 | 5.63 | -0.37 | 19.40 |
| Lig^DH^ | 6.03 | 49.81 | 43.22-50.73 | PZE_106041753 | PZE_106051230 | 6.26 | -0.39 | 23.50 |
| Lig^haploid^ | 6.04 | 69.62 | 65.63-71.16 | PZE_106064885 | PZE_106065229 | 4.20 | -0.44 | 12.90 |
| Cel^haploid^ | 1.08 | 54.19 | 54.1-54.20 | PZE_101177728 | PZE_101241459 | 12.08 | -1.53 | 24.10 |
| Cel^haploid^ | 2.02 | 43.60 | 37.97-44.03 | SYN2695 | PZE_102019300 | 3.47 | 0.82 | 6.60 |
| Cel^DH^ | 2.02 | 55.31 | 53.64-60.35 | PZE_102030524 | PZE_102039914 | 5.74 | 1.13 | 12.90 |
| Cel^haploid^ | 3.06 | 147.65 | 146.98-149.31 | PZE_103151399 | PZE_103165581 | 4.12 | 0.92 | 8.30 |
| Cel^DH^ | 5.06 | 133.84 | 133.61-134.33 | PZE_105149244 | PZE_105142633 | 4.87 | -1.14 | 12.40 |
| Cel^haploid^ | 7.04 | 102.81 | 97.86-106.65 | PZE_107105855 | SYN3390 | 5.59 | -1.13 | 10.60 |
| Cel^DH^ | 8.04 | 78.11 | 77.75-78.48 | PZE_108060911 | SYN37439 | 3.58 | -1.10 | 13.30 |
| Cel^haploid^ | 9.06 | 109.97 | 108.29-115.21 | SYN25121 | PZE_109104633 | 3.33 | -0.78 | 5.70 |
| WSC^DH^ | 1.04 | 56.99 | 56.42-57.53 | PZE_101179982 | SYN36407 | 5.69 | 1.79 | 14.50 |
| WSC^DH^ | 6.03 | 51.65 | 50.73-53.40 | PZE_106049961 | PZE_106052536 | 3.85 | -1.46 | 9.50 |
| WSC^haploid^ | 8.05 | 85.38 | 84.79-85.70 | PZE_108069355 | PZE_108074750 | 4.88 | 1.74 | 14.60 |
| WSC^DH^ | 8.05 | 94.79 | 94.47-95.20 | PZE_108077037 | PZE_108076067 | 7.93 | 2.23 | 20.80 |
|  |  |  |  |  |  |  |  |  |

**^a^**The peak position with the highest LOD of each QTL.

^b^The Flanking markers of the identified QTL according to B73 reference sequence Version 5.60.

^c^Estimate of allele effect.

^DH^ QTL detected in DH population.

^haploid^QTL detected in Haploid population.

QTL shown in one frame represented that the genetic distance between them was less than 20cM.

**Table S5 Annotation of the 45 predicted genes located within putative RPR QTL interval in DH population.**

| **Gene ID** | **Start Position^a^** | **Description** |
| --- | --- | --- |
| GRMZM5G814057 | 176271377 | Transmembrane 9 superfamily member, putative, expressed |
| GRMZM2G056505 | 176326899 | CPuORF19 - conserved peptide uORF-containing transcript, expressed |
| GRMZM2G056629 | 176347442 | Aldose 1-epimerase, putative, expressed |
| GRMZM2G075637 | 176387128 | SET, putative, expressed |
| GRMZM2G081589 | 176487861 | Sucrose transporter BoSUT1, putative, expressed |
| GRMZM2G138365 | 176549610 | Unknown |
| GRMZM2G156734 | 176587988 | Znc finger, C3HC4 type, putative, expressed |
| GRMZM2G132706 | 176610086 | Cytokinin-O-glucosyltransferase 2, putative, expressed |
| AC201791.3_FG005 | 176788138 | Unknown |
| GRMZM5G870219 | 176799407 | Oxygen evolving enhancer protein 3, identical, putative, expressed |
| GRMZM2G457262 | 176828886 | Unknown |
| GRMZM5G827556 | 176828886 | Unknown |
| GRMZM2G129929 | 176866260 | Unknown |
| GRMZM2G325683 | 176870667 | Unknown |
| GRMZM2G325693 | 176876092 | Unknown |
| GRMZM2G027193 | 176895071 | Unknown |
| GRMZM2G146380 | 176939481 | No apical meristem protein, putative, expressed |
| GRMZM2G001223 | 176991826 | MYB family transcription factor, putative, expressed |
| AC190990.2_FG005 | 177016937 | DNA replication |
| GRMZM2G099989 | 177123526 | Unknown |
| GRMZM2G316366 | 177222141 | OsMADS27 - MADS-box family gene with MIKCc type-box, expressed |
| GRMZM2G492156 | 177229409 | OsMADS27 - MADS-box family gene with MIKCc type-box, expressed |
| GRMZM2G016707 | 177233625 | Unknown |
| AC210598.3_FG003 | 177239242 | Unknown |
| GRMZM2G103152 | 177442845 | Unknown |
| GRMZM2G454702 | 177485913 | Unknown |
| GRMZM5G801651 | 177662568 | Choline transporter-related, putative, expressed |
| GRMZM5G896337 | 177663621 | Choline transporter-related, putative, expressed |
| GRMZM2G459166 | 177668908 | OsFBX77 - F-box domain containing protein, expressed |
| GRMZM2G086246 | 177739535 | Unknown |
| GRMZM5G861269 | 177745636 | Dynein light chain type 1 family protein, putative, expressed |
| GRMZM2G410710 | 177824670 | Mitochondrial prohibitin complex protein 1, putative, expressed |
| GRMZM2G176382 | 177830186 | Unknown |
| GRMZM2G176253 | 177840203 | Peptide transporter PTR2, putative, expressed |
| AC212286.3_FG007 | 177973750 | Unknown |
| GRMZM2G095861 | 177975509 | Potassium channel AKT1, putative, expressed |
| GRMZM2G095782 | 178036903 | tRNApseudouridine synthase family protein, putative, expressed |
| GRMZM5G884270 | 178073884 | Phosphatidylinositolglycan class N family protein, expressed |
| GRMZM2G377168 | 178086882 | Photosystem II 5 kDa protein, chloroplast precursor, putative, expressed |
| GRMZM2G077612 | 178090178 | Unknown |
| GRMZM2G444643 | 178116669 | ASC1, putative, expressed |
| GRMZM2G433002 | 178235052 | Hydrolase, alpha/beta fold family domain containing protein, expressed |
| GRMZM2G131746 | 178240933 | Unknown |
| GRMZM2G131727 | 178243013 | N-terminal protein myristoylation; |
| GRMZM2G060355 | 178263018 | Unknown |

^a^Nucleotide position in the B73 reference sequence (version 5b.60; MaizeSequence, <http://www.maizesequence.org/>)

**Table S6 Annotation of the 191 predicted genes located within putative RPR QTL interval in haploid population.**

| Gene ID | Start Position^a^ | Description |
| --- | --- | --- |
| GRMZM2G032554 | 13879463 | Expressed protein |
| GRMZM2G032583 | 13884934 | Unknown |
| GRMZM2G100732 | 13986968 | DNA-binding protein, putative, expressed |
| GRMZM2G169553 | 14106651 | Purine permease, putative, expressed |
| GRMZM2G162447 | 14178945 | Invertase/pectin methylesterase inhibitor family protein, putative |
| GRMZM2G024312 | 14205645 | Zinc finger, C3HC4 type domain containing protein, expressed |
| GRMZM2G322817 | 14222092 | Unknown |
| GRMZM2G024517 | 14229619 | Unknown |
| GRMZM2G163067 | 14266571 | Unknown |
| GRMZM2G098099 | 14293505 | Unknown |
| GRMZM2G014005 | 14322542 | Unknown |
| GRMZM2G014009 | 14331192 | Unknown |
| GRMZM2G014187 | 14335492 | Regulatory protein, putative, expressed |
| GRMZM2G066489 | 14397636 | Glutamate receptor, putative, expressed |
| GRMZM2G405064 | 14433162 | CAPIP1, putative, expressed |
| GRMZM2G010011 | 14438836 | Zinc finger family protein, putative, expressed |
| GRMZM2G146847 | 14462634 | RING-H2 finger protein ATL2A, putative, expressed |
| GRMZM2G146866 | 14466612 | Protein kinase domain containing protein, expressed |
| GRMZM2G016705 | 14526287 | Expressed protein |
| GRMZM2G314386 | 14530124 | Expressed protein |
| GRMZM2G017197 | 14534118 | Unknown |
| GRMZM2G314396 | 14534118 | CAMK_CAMK_like.27 - CAMK includes calcium/calmodulindepedent protein kinases |
| GRMZM2G314412 | 14545405 | U-box domain-containing protein, putative, expressed |
| GRMZM2G479665 | 14613507 | Protein phosphatase 2C, |
| GRMZM2G479684 | 14620170 | Core histone H2A/H2B/H3/H4 domain containing protein, putative |
| GRMZM2G054130 | 14660441 | Oral cancer overexpressed protein 1, putative, expressed |
| GRMZM2G318527 | 14679417 | ZR1 protein, putative, expressed |
| GRMZM2G421256 | 14714775 | MYB family transcription factor |
| GRMZM2G421279 | 14720962 | Core histone H2A/H2B/H3/H4 domain containing protein |
| GRMZM2G120717 | 14722000 | Oral cancer overexpressed protein |
| GRMZM2G120724 | 14728687 | Expansin precursor |
| GRMZM2G356273 | 14755031 | Expansin precursor |
| GRMZM2G076844 | 14798721 | Expressed protein |
| GRMZM2G076771 | 14804053 | PPR repeat domain containing protein, putative, expressed |
| GRMZM2G303147 | 14822431 | Unknown |
| GRMZM2G303118 | 14825894 | tRNA-splicing endonuclease positive effector-related |
| AC191961.3_FG009 | 14869123 | tRNA-splicing endonuclease positive effector-related |
| GRMZM2G129386 | 14882783 | SIT4 phosphatase-associated protein domain containing protein |
| GRMZM2G430500 | 14891652 | Unknown |
| GRMZM2G129399 | 14892972 | Heavy metal-associated domain containing protein |
| GRMZM2G129450 | 14896479 | Unknown |
| GRMZM2G402837 | 15000058 | OsMADS18 - MADS-box family gene with MIKCc type-box |
| GRMZM2G102059 | 15022549 | B3 DNA binding domain containing protein |
| AC186904.4_FG007 | 15114411 | Unknown |
| GRMZM2G050262 | 15123576 | Unknown |
| GRMZM2G050234 | 15127944 | Naringenin,2-oxoglutarate 3-dioxygenase |
| GRMZM2G050216 | 15132700 | Zinc finger, C3HC4 type domain containing protein |
| GRMZM2G045398 | 15140829 | Zinc finger, C3HC4 type domain containing protein |
| GRMZM2G464772 | 15192689 | Cytokinin inducible protein, putative |
| GRMZM2G038835 | 15243138 | Unknown |
| GRMZM2G038851 | 15245261 | Ubiquitin-conjugating enzyme |
| GRMZM2G341089 | 15266020 | Ubiquitin-conjugating enzyme |
| GRMZM2G109869 | 15340482 | GRAS family transcription factor domain containing protein |
| GRMZM2G702945 | 15355908 | Unknown |
| GRMZM2G702948 | 15478269 | Unknown |
| GRMZM2G318408 | 15481802 | Zinc finger, C3HC4 type domain containing protein |
| GRMZM2G426067 | 15567011 | Unknown |
| GRMZM2G125494 | 15570785 | Uncharacterized mscS family protein, |
| AC217385.3_FG009 | 15582700 | Unknown |
| GRMZM2G153378 | 15682289 | Unknown |
| GRMZM2G164318 | 15865938 | Fatty acid hydroxylase |
| GRMZM2G164340 | 15872003 | Unknown |
| GRMZM2G164378 | 15881088 | Unknown |
| GRMZM2G164405 | 15889503 | Aminotransferase, classes I and II, domain containing protein |
| GRMZM2G164413 | 15895715 | Unknown |
| GRMZM2G164428 | 15908515 | Unknown |
| GRMZM2G114131 | 15967914 | DeoxyribonucleasetatD |
| GRMZM2G114186 | 15973106 | Unknown |
| GRMZM2G114192 | 15976524 | RhoGAP domain containing protein |
| GRMZM2G114220 | 15984741 | Ubiquitin fusion degradation protein |
| GRMZM2G114276 | 15993945 | Leucine-rich repeat family protein |
| GRMZM2G076766 | 16010167 | Unknown |
| GRMZM2G080156 | 16023592 | 3-oxo-5-alpha-steroid 4-dehydrogenase |
| GRMZM2G080274 | 16036480 | Histone H1 |
| GRMZM2G556131 | 16107968 | Dihydrodipicolinate synthase, chloroplast precursor |
| GRMZM2G106921 | 16123074 | C4-dicarboxylate transporter/malic acid transport protein domain containing protein |
| GRMZM2G106881 | 16125764 | ZOS4-12 - C2H2 zinc finger protein |
| GRMZM2G003752 | 16219232 | Fasciclin-like arabinogalactan protein membrane |
| GRMZM2G303135 | 16233441 | Exostosin family domain containing protein |
| GRMZM2G476597 | 16293302 | Cytochrome P450, putative |
| GRMZM2G175728 | 16302477 | Copper chaperone for superoxide dismutase |
| GRMZM2G086210 | 16342319 | HOTHEAD precursor, putative |
| GRMZM2G086430 | 16348456 | Uncharacterized membrane protein |
| AC191654.3_FG004 | 16355785 | Unknown |
| GRMZM2G086484 | 16361016 | Unknown |
| GRMZM2G161902 | 16398790 | Unknown |
| GRMZM2G161913 | 16450273 | Tetratricopeptide repeat domain containing protein |
| GRMZM2G042756 | 16532570 | Dehydration-responsive element-binding protein, putative |
| GRMZM2G368838 | 16539933 | AP2 domain containing protein |
| GRMZM2G068128 | 16546854 | RING-H2 finger protein |
| GRMZM2G080992 | 16638278 | MATE efflux family protein |
| GRMZM2G081032 | 16654256 | OsFBX148 - F-box domain containing protein |
| GRMZM2G081060 | 16658315 | Zinc RING finger protein |
| GRMZM2G128179 | 16675700 | Dehydration response related protein |
| GRMZM2G318956 | 16769117 | Unknown |
| GRMZM2G069525 | 16870336 | MYB family transcription factor |
| GRMZM2G039455 | 16938974 | PINHEAD |
| GRMZM2G129261 | 17094412 | ZOS4-11 - C2H2 zinc finger protein |
| GRMZM2G117060 | 17250064 | SNF2 family N-terminal domain containing protein |
| AC210678.3_FG004 | 17259392 | Unknown |
| GRMZM2G176647 | 17334303 | Transducin/WD40 repeat-like superfamily protein |
| GRMZM2G145968 | 17348097 | HMG1/2 |
| GRMZM2G057131 | 17430807 | RWP-RK domain-containing protein |
| GRMZM2G138886 | 17476257 | Cyclin |
| GRMZM2G082181 | 17543996 | Unknown |
| GRMZM2G384126 | 17555876 | Unknown |
| GRMZM2G142672 | 17560385 | Unknown |
| GRMZM2G142664 | 17580338 | Growth regulator related protein |
| GRMZM2G041506 | 17607193 | Unknown |
| GRMZM2G410728 | 17676008 | Unknown |
| GRMZM2G109865 | 17689071 | Transmembrane amino acid transporter protein |
| GRMZM2G101333 | 17788654 | GDSL-like lipase/acylhydrolase, |
| GRMZM2G101383 | 17791589 | Pseudouridine synthase family protein |
| GRMZM2G101408 | 17796828 | Coiled-coil domain-containing protein 124 putative expressed |
| GRMZM5G834171 | 17857003 | Unknown |
| GRMZM2G322819 | 17861166 | OsPOP9 - Putative Prolyl Oligopeptidase homologue |
| GRMZM2G027891 | 17867648 | Mechanosensitive channel of small conductance-like 5 |
| GRMZM2G088601 | 17901565 | Unknown |
| GRMZM2G019783 | 17986668 | ATFUC1 |
| GRMZM2G019863 | 17999113 | Amine oxidase |
| GRMZM2G019876 | 18003198 | Unknown |
| GRMZM2G020500 | 18073161 | Cytochrome P450 |
| GRMZM2G175504 | 18113572 | STE_MEKK_ste11_MAP3K.17 - STE kinases include homologs to sterile 7, sterile 11 and sterile 20 from |
| GRMZM2G174807 | 18133344 | Aquaporin protein, putative |
| GRMZM2G047347 | 18202741 | Unknown |
| GRMZM2G349344 | 18203836 | Protein kinase |
| GRMZM2G388585 | 18241594 | Expressed protein |
| GRMZM2G087678 | 18247490 | ATROPGEF7/ROPGEF7 |
| GRMZM2G163110 | 18308012 | OsSub44 - Putative Subtilisin homologue |
| GRMZM2G462986 | 18316891 | OsSub44 - Putative Subtilisin homologue |
| GRMZM2G036996 | 18350561 | Unknown |
| GRMZM2G172795 | 18417370 | Anthocyanin regulatory Lc protein |
| GRMZM2G168474 | 18460858 | Cis-zeatin O-glucosyltransferase |
| GRMZM2G168516 | 18466456 | Unknown |
| GRMZM2G399862 | 18507575 | Unknown |
| GRMZM2G099130 | 18509782 | Unknown |
| GRMZM2G144153 | 18615568 | Glutathione peroxidase domain containing protein |
| GRMZM2G144083 | 18620426 | Copper-transporting ATPase 3 |
| GRMZM2G120016 | 18644060 | Cis-zeatin O-glucosyltransferase |
| GRMZM2G143512 | 18705084 | Copper-transporting ATPase 3 |
| GRMZM2G171556 | 18762222 | Serine racemase |
| GRMZM2G469313 | 18766156 | Receptor-like protein kinase 5 precursor |
| GRMZM2G071253 | 18811363 | Zinc knuckle domain containing protein |
| GRMZM2G071327 | 18815692 | Actin-depolymerizing factor |
| AC214523.3_FG002 | 18984281 | Unknown |
| GRMZM2G044143 | 18987884 | Beige/BEACH domain containing protein |
| GRMZM2G003307 | 19037250 | Pre-mRNA-processing factor 31 |
| GRMZM5G825321 | 19145889 | Scarecrow, putative |
| GRMZM2G162271 | 19195367 | Unknown |
| GRMZM2G162276 | 19199595 | LTPL122 - Protease inhibitor/seed storage/LTP family protein precursor |
| GRMZM2G162319 | 19251973 | Unknown |
| GRMZM2G462258 | 19253013 | Beta-amylase |
| GRMZM2G462261 | 19257350 | Glycosyl transferase 8 domain containing protein |
| GRMZM2G162333 | 19264742 | Pectinesterase |
| GRMZM2G077811 | 19281986 | Thioesterase family protein |
| GRMZM2G170509 | 19319916 | Thioesterase family protein |
| GRMZM2G170516 | 19323633 | Unknown |
| GRMZM2G170520 | 19359170 | ZOS4-10 - C2H2 zinc finger protein |
| GRMZM2G170522 | 19365673 | Unknown |
| GRMZM2G132756 | 19431051 | ZOS4-10 - C2H2 zinc finger protein |
| GRMZM2G132774 | 19438615 | Cyclin |
| GRMZM2G132777 | 19443026 | Amino acid kinase |
| GRMZM2G458974 | 19451472 | 40S ribosomal protein S7 |
| GRMZM2G158657 | 19458968 | Carotenoid cleavage dioxygenase 7, chloroplast precursor |
| GRMZM2G084062 | 19602388 | Eukaryotic translation initiation factor 3 subunit C |
| GRMZM2G129300 | 19713890 | Unknown |
| GRMZM2G129298 | 19721489 | Unknown |
| GRMZM2G121275 | 19828184 | Aquaporin protein |
| GRMZM2G121223 | 19832065 | START domain containing protein putative expressed |
| GRMZM2G702954 | 19909592 | Unknown |
| GRMZM2G101290 | 19914845 | Lactate/malate dehydrogenase |
| GRMZM2G101181 | 19917630 | Growth regulator related protein |
| GRMZM2G078648 | 19989571 | Unknown |
| GRMZM2G172936 | 20008480 | AP2 domain containing protein |
| GRMZM2G411639 | 20046124 | Peptide chain release factor protein |
| GRMZM2G028969 | 20096078 | AP2 domain containing protein |
| GRMZM2G325513 | 20102238 | DREB subfamily A-4 of ERF/AP2 transcription factor family |
| AC207188.3_FG002 | 20119937 | Unknown |
| GRMZM2G105137 | 20122023 | MYB family transcription factor |
| GRMZM2G139458 | 20192163 | Unknown |
| GRMZM2G139463 | 20195602 | L-asparaginase precursor protein |
| GRMZM2G429035 | 20362638 | Unknown |
| GRMZM2G044884 | 20393500 | Cytokinin-N-glucosyltransferase 1 |
| GRMZM2G044752 | 20400706 | Homeobox associated leucine zipper |
| GRMZM2G347027 | 20407553 | Rhodanese-like domain containing protein |
| GRMZM2G346982 | 20418891 | Unknown |
| GRMZM2G328781 | 20522957 | Inactive receptor kinase At2g26730 precursor |
| GRMZM2G026050 | 20529696 | Ethylene-responsive element-binding |
| GRMZM2G175816 | 20536334 | Hydrolase, NUDIX family, domain containing protein |
| GRMZM2G475678 | 20563651 | AP2 domain containing protein |
| GRMZM5G805505 | 20607306 | AP2 domain containing protein |

^a^Nucleotide position in the B73 reference sequence (version 5b.60; MaizeSequence, <http://www.maizesequence.org/>)

**Table S7 Annotation of the 24 predicted genes located within the putative IVDMD QTL interval in DH population.**

| **Gene ID** | **Start Position^a^** | **Description** |
| --- | --- | --- |
| AC199217.3_FG004 | 120316735 | Unknown |
| GRMZM2G436864 | 120363477 | Unknown |
| GRMZM2G088336 | 120458694 | Unknown |
| AC187099.5_FG005 | 120576304 | Zinc finger RING-type putative expressed |
| GRMZM2G332410 | 120592109 | Unknown |
| GRMZM2G033230 | 120608901 | (bZIP19) Basic-leucine zipper (bZIP) transcription factor family protein |
| GRMZM2G173195 | 120753225 | (GPDHC1) 6-phosphogluconate dehydrogenase family protein |
| GRMZM2G380561 | 120768105 | (BG1) Beta-1 3-glucanase 1 |
| GRMZM2G083016 | 120773908 | (AMC6, ATMC5, ATMCP2B, MC5) Metacaspase 5 |
| GRMZM2G066041 | 120870569 | (AMC6, ATMC5, ATMCP2B, MC5) Metacaspase 5 |
| GRMZM2G066158 | 120874781 | (ATERF12, ERF12) ERF domain protein 12 |
| GRMZM2G066204 | 120877364 | Unknown |
| GRMZM2G174347 | 120960110 | (ATERF-9, ATERF9, ERF9) Erf domain protein 9 |
| GRMZM2G473111 | 120963905 | Unknown |
| GRMZM2G089355 | 121130629 | Zinc finger C3HC4 type family protein putative expressed |
| GRMZM2G388371 | 121158301 | Ribosomal protein L11 methyltransferase-related |
| GRMZM2G179444 | 121177893 | Pectin lyase-like superfamily protein |
| GRMZM2G479869 | 121206618 | Nucleotide/sugar transporter family protein |
| GRMZM2G410318 | 121283721 | (ATMSH5, MSH5) MUTS-homologue 5 |
| GRMZM2G043069 | 121418738 | Protein kinase superfamily protein |
| GRMZM2G042865 | 121435529 | (DOGT1, UGT73C5) Don-glucosyltransferase 1 |
| GRMZM2G074631 | 121484561 | (UGT73B3) UDP-glucosyl transferase 73B3 |
| GRMZM2G027530 | 121579977 | Unknown |
| GRMZM2G010927 | 121669230 | HSP20-like chaperones superfamily protein |

^a^Nucleotide position in the B73 reference sequence (version 5b.60; MaizeSequence, <http://www.maizesequence.org/>)

**Table S8 Annotation of the 191 predicted genes located within putative IVDMD QTL interval in haploid population.**

| Gene ID | Start Position^a^ | Description |
| --- | --- | --- |
| GRMZM2G043069 | 121418738 | Protein kinase superfamily protein |
| GRMZM2G042865 | 121435529 | (DOGT1, UGT73C5) Don-glucosyltransferase 1 |
| GRMZM2G074631 | 121484561 | (UGT73B3) UDP-glucosyl transferase 73B3 |
| GRMZM2G027530 | 121579977 | Unknown |
| GRMZM2G010927 | 121669230 | HSP20-like chaperones superfamily protein |
| GRMZM2G366873 | 121797016 | Auxin-responsive GH3 family protein |
| GRMZM2G082792 | 121883080 | (FQR1) Flavodoxin-like quinone reductase 1 |
| GRMZM2G384755 | 121889087 | Serine/threonine-protein kinase receptor precursor putative expressed |
| GRMZM2G082894 | 121901413 | Unknown |
| GRMZM2G026452 | 122003962 | Unknown |
| GRMZM2G100809 | 122031317 | (SC3) Secretory carrier 3 |
| GRMZM2G402137 | 122040827 | NAF1 domain containing protein expressed |
| GRMZM2G157631 | 122060823 | OsFBX169 - F-box domain containing protein expressed |
| GRMZM2G087426 | 122110472 | Unknown |
| GRMZM2G060327 | 122141815 | Ubiquitin-specific protease 25 |
| GRMZM2G060284 | 122146888 | Transcription initiation factor IIF beta subunit |
| GRMZM2G096600 | 122221082 | Tesmin/TSO1-like CXC domain containing protein, expressed |
| GRMZM2G096553 | 122225283 | Pseudouridylate synthase, putative, expressed |
| GRMZM2G173198 | 122238600 | Tesmin/TSO1-like CXC domain containing protein expressed |
| GRMZM2G173137 | 122240358 | Pseudouridylate synthase, putative, expressed |
| GRMZM2G060213 | 122408765 | Unknown |
| GRMZM2G527250 | 122422960 | Unknown |
| GRMZM2G074648 | 122426675 | Calreticulin precursor protein, putative, expressed |
| GRMZM2G375222 | 122458204 | Transcription elongation factor SPT5 homolog 1, putative, expressed |
| GRMZM2G154936 | 122751582 | Glucan endo-1,3-beta-glucosidase-like protein 3 precursor, putative, expressed |
| GRMZM2G035944 | 122903771 | TCP family transcription factor, putative, expressed |
| GRMZM2G035651 | 122928857 | NLI interacting factor-like phosphatase, putative, expressed |
| AC209819.3_FG012 | 122948949 | Ras-related protein, putative, expressed |
| GRMZM5G811797 | 122951333 | Ras-related protein, putative, expressed |
| AC209819.3_FG010 | 122954415 | Unknown |
| AC209819.3_FG003 | 123004028 | Heat- and acid-stable phosphoprotein, putative, expressed |
| AC209819.3_FG005 | 123041911 | O-methyltransferase, putative, expressed |
| AC209819.3_FG006 | 123055027 | Myosin heavy chain-related, putative, expressed |
| AC209819.3_FG007 | 123057720 | Unknown |
| AC209819.3_FG009 | 123061033 | Dof zinc finger domain containing protein, putative, expressed |
| AC209819.3_FG008 | 123063066 | Unknown |
| GRMZM2G096115 | 123145419 | Encodes CBL-interacting protein kinase 12 (CIPK12). |
| GRMZM2G095955 | 123149285 | Unknown |
| GRMZM2G091592 | 123168303 | Unknown |
| GRMZM5G850924 | 123253292 | Unknown |
| AC234149.1_FG002 | 123322670 | Unknown |
| AC234149.1_FG001 | 123324138 | Unknown |
| GRMZM2G073044 | 123469338 | Unknown |
| GRMZM2G179264 | 123501085 | FT, together with LFY |
| GRMZM2G179274 | 123507161 | Unknown |
| GRMZM2G479987 | 123509007 | Unknown |
| GRMZM2G068943 | 123601206 | Trehalose-6-phosphate synthase, putative, expressed |
| AC187243.3_FG005 | 123614699 | Unknown |
| GRMZM2G180668 | 123810520 | VQ motif-containing protein |
| GRMZM2G104283 | 123844264 | Protein serine/threonine/tyrosine kinase activity |
| GRMZM2G350023 | 123899080 | Unknown |
| GRMZM2G136369 | 123906214 | Homeobox domain containing protein, expressed |
| GRMZM2G122727 | 123928358 | Unknown |
| GRMZM5G891056 | 123944098 | Unknown |
| AC233864.1_FG002 | 124013979 | Unknown |
| GRMZM5G892365 | 124033198 | Unknown |
| AC233864.1_FG009 | 124042906 | Unknown |
| AC233864.1_FG014 | 124086876 | Unknown |
| GRMZM2G115346 | 124144400 | Unknown |
| GRMZM2G415891 | 124164647 | Unknown |
| GRMZM2G115364 | 124165959 | Unknown |
| GRMZM2G169236 | 124188697 | Unknown |
| GRMZM2G162884 | 124208874 | Unknown |
| GRMZM2G084477 | 124223433 | Unknown |
| GRMZM2G127844 | 124282351 | Peptidyl-tRNA hydrolase, putative, expressed |
| GRMZM2G135381 | 124357134 | GATA zinc finger domain containing protein, expressed |
| GRMZM2G038082 | 124433434 | Unknown |
| GRMZM2G330693 | 124434479 | Unknown |
| GRMZM5G802899 | 124437928 | Unknown |
| GRMZM2G173700 | 124457224 | Kinesin motor protein-related, putative, expressed |
| GRMZM2G173710 | 124485354 | Histidine-containing phosphotransfer protein, putative, expressed |
| GRMZM2G071223 | 124583741 | Unknown |
| GRMZM2G071339 | 124587539 | Pectinesterase, putative, expressed |
| GRMZM2G163546 | 124622026 | Unknown |
| GRMZM2G058690 | 124645482 | IQ calmodulin-binding motif family protein, putative, expressed |
| GRMZM2G058745 | 124652438 | Hexokinase, putative, expressed |
| GRMZM2G109842 | 124707856 | Profilin domain containing protein, expressed |
| GRMZM2G106819 | 124796660 | Tolerance to heavy metals and oxidative stress |
| GRMZM2G106917 | 124799105 | Unknown |
| GRMZM2G093418 | 124839636 | Unknown |
| GRMZM2G093404 | 124841672 | Zinc finger/CCCH transcription factor, putative, expressed |
| GRMZM2G095807 | 124847602 | Anthocyanidin 5,3-O-glucosyltransferase, putative, expressed |
| GRMZM2G082707 | 124930000 | 50S ribosomal protein L20, putative, expressed |
| GRMZM2G319747 | 124936785 | Unknown |
| GRMZM2G015433 | 124950950 | WRKY23, expressed |
| GRMZM2G353822 | 125007017 | Unknown |
| GRMZM5G855375 | 125007024 | Unknown |
| GRMZM2G052200 | 125008668 | Unknown |
| GRMZM2G408989 | 125111092 | ER lumen protein retaining receptor, putative, expressed |
| AC205471.4_FG003 | 125180331 | HSF-type DNA-binding domain containing protein, expressed |
| AC205471.4_FG008 | 125196525 | TOO MANY MOUTHS precursor, putative, expressed |
| AC205471.4_FG007 | 125225855 | Unknown |
| GRMZM5G861659 | 125261081 | INVOLVED IN: positive regulation of flower development; LOCATED IN: nucleus; |
| GRMZM2G044866 | 125294565 | Unknown |
| GRMZM2G044797 | 125299768 | Unknown |
| GRMZM2G044771 | 125305205 | Unknown |
| GRMZM2G044744 | 125308562 | Starch synthase, putative, expressed |
| GRMZM2G420684 | 125392109 | Unknown |
| GRMZM2G121117 | 125417802 | Unknown |
| GRMZM2G048117 | 125466267 | Unknown |
| GRMZM2G048200 | 125469959 | GDSL-like lipase/acylhydrolase, putative, expressed |
| AC187826.3_FG007 | 125522018 | Unknown |
| GRMZM2G017142 | 125536101 | PHD finger protein, putative, expressed |
| GRMZM2G029027 | 125589568 | Arginyl-tRNAsynthetase, putative, expressed |
| GRMZM2G034551 | 125741136 | Metal cation transporter, putative, expressed |
| GRMZM2G059693 | 125902255 | Lipase, putative, expressed |
| GRMZM5G855411 | 125974327 | Unknown |
| GRMZM2G380515 | 126076746 | Unknown |
| AC210792.5_FG003 | 126142833 | Unknown |
| GRMZM2G163542 | 126206978 | Unknown |
| GRMZM2G118834 | 126280300 | Unknown |
| GRMZM5G804181 | 126296004 | Unknown |
| AC210413.3_FG005 | 126400002 | Unknown |
| GRMZM5G873249 | 126441194 | Unknown |
| GRMZM5G871000 | 126443109 | Unknown |
| AC208327.4_FG002 | 126449851 | OsGrx_S15.1 - glutaredoxin subgroup II, expressed |
| AC208327.4_FG003 | 126490922 | Unknown |
| AC208327.4_FG006 | 126549890 | N-acetylglucosaminyltransferase, putative, expressed |
| GRMZM2G389118 | 126595171 | An integral membrane GTPase |
| GRMZM2G389015 | 126599841 | Unknown |
| AC197021.3_FG004 | 126620838 | Unknown |
| GRMZM2G088020 | 126626745 | Unknown |
| GRMZM2G150367 | 126699195 | Stress-related protein, putative, expressed Rubber elongation factor protein (REF) |
| GRMZM2G150434 | 126701828 | Peripheral-type benzodiazepine receptor, putative, expressed |
| GRMZM2G150453 | 126726262 | Unknown |
| GRMZM2G166089 | 126868440 | Cullin, putative, expressed |
| GRMZM2G467497 | 126880182 | Prolinesynthetase co-transcribed bacterial homolog protein putative expressed |
| GRMZM2G141066 | 126901418 | Unknown |
| GRMZM2G381129 | 126953822 | Prolinesynthetase co-transcribed bacterial homolog protein putative expressed |
| GRMZM2G050800 | 126999827 | Serine/threonine-protein phosphatase 2A regulatory subunit B subunitgamma |
| GRMZM2G120178 | 127072692 | Serine/threonine-protein phosphatase 2A regulatory subunit B subunitgamma |
| GRMZM2G135978 | 127112957 | OsFBL21 - F-box domain and LRR containing protein, expressed |
| GRMZM2G090963 | 127335528 | Unknown |
| GRMZM2G150485 | 127487910 | Bifunctional protein folD, putative, expressed |
| GRMZM2G135320 | 127621662 | Unknown |
| GRMZM2G135366 | 127625737 | Unknown |
| GRMZM2G150474 | 127719984 | Glutathione S-transferase, putative, expressed |
| GRMZM2G025954 | 127775718 | Unknown |
| GRMZM2G443340 | 127853411 | Unknown |
| GRMZM2G139434 | 127881302 | Unknown |
| GRMZM2G372200 | 127959986 | BRCT domain-containing DNA repair protein |
| AC200884.4_FG002 | 128124567 | Unknown |
| GRMZM2G011491 | 128128073 | PPR repeat domain containing protein, putative, expressed |
| GRMZM2G053952 | 128203219 | Unknown |
| GRMZM2G355389 | 128206303 | Unknown |
| GRMZM2G054040 | 128246413 | CRS1/YhbY domain containing protein, putative, expressed |
| AC225346.3_FG001 | 128343673 | Unknown |
| GRMZM2G170798 | 128348231 | Serine/threonine protein phosphatase, putative, expressed |
| GRMZM2G111306 | 128427935 | Myb-like DNA-binding domain, SHAQKYF class family protein, expressed |
| GRMZM5G816304 | 128433773 | Methyltransferase domain containing protein, putative, expressed |
| GRMZM2G037350 | 128481827 | WD domain, G-beta repeat domain containing protein, expressed |
| GRMZM5G876434 | 128486678 | WD domain, G-beta repeat domain containing protein, expressed |
| GRMZM2G700655 | 128542778 | Unknown |
| GRMZM2G165930 | 128552327 | DNA binding protein putative expressed |
| GRMZM5G834260 | 128602397 | Unknown |
| GRMZM5G817255 | 128698509 | RNA recognition motif containing protein, putative, expressed |
| AC202915.3_FG002 | 128751312 | Phosphoglycerate mutase, putative, expressed |
| GRMZM2G399541 | 128811372 | Unknown |
| GRMZM2G073842 | 128916712 | Unknown |
| GRMZM2G171781 | 129072528 | MYB family transcription factor, putative, expressed |
| GRMZM2G013450 | 129119355 | Mitochondrial carrier protein, putative, expressed |
| GRMZM2G121510 | 129290690 | Unknown |
| GRMZM2G083292 | 129341989 | Unknown |
| GRMZM2G323415 | 129386628 | Unknown |
| GRMZM2G002361 | 129409767 | Unknown |
| GRMZM2G002427 | 129414617 | Unknown |
| GRMZM2G042593 | 129496306 | IF, putative, expressed |
| GRMZM2G343024 | 129500966 | Protein kinase, putative, expressed |
| GRMZM2G117198 | 129620163 | S-adenosylmethioninesynthetase, putative, expressed |
| GRMZM2G383215 | 129651033 | Cyclin-dependent kinase B2-1, putative, expressed |
| GRMZM2G084369 | 129692395 | Unknown |
| GRMZM5G826666 | 129696124 | Unknown |

^a^Nucleotide position in the B73 reference sequence (version 5b.60; MaizeSequence, <http://www.maizesequence.org/>)
